# Exploiting spatiotemporal regulation of FZD5 during neural patterning for efficient ventral midbrain specification

**DOI:** 10.1242/dev.202545

**Published:** 2024-03-04

**Authors:** Andy Yang, Rony Chidiac, Emma Russo, Hendrik Steenland, Quinn Pauli, Robert Bonin, Levi L. Blazer, Jarrett J. Adams, Sachdev S. Sidhu, Aleksandrina Goeva, Ali Salahpour, Stephane Angers

**Affiliations:** ^1^ Donnelly Centre for Cellular and Biomolecular Research, University of Toronto, Toronto, ON M5S 3E1, Canada; ^2^ Leslie Dan Faculty of Pharmacy, University of Toronto, Toronto, ON M5S 3M2, Canada; ^3^ Department of Pharmacology and Toxicology, Temerty Faculty of Medicine, University of Toronto, Toronto, ON M5S 1A8, Canada; ^4^ NeuroTek Innovative Technology, Toronto, ON M6C 3A2, Canada; ^5^ Department of Molecular Genetics, Temerty Faculty of Medicine, University of Toronto, Toronto, ON M5S 1A8, Canada; ^6^ Broad Institute of Massachusetts Institute of Technology and Harvard, Cambridge, MA 02142, USA; ^7^ Department of Biochemistry, Temerty Faculty of Medicine, University of Toronto, Toronto, ON M5S 1A8, Canada

**Keywords:** Wnt signaling, Stem cell differentiation, Neural patterning, Dopaminergic neuron, Frizzled receptors

## Abstract

The Wnt/β-catenin signaling governs anterior-posterior neural patterning during development. Current human pluripotent stem cell (hPSC) differentiation protocols use a GSK3 inhibitor to activate Wnt signaling to promote posterior neural fate specification. However, GSK3 is a pleiotropic kinase involved in multiple signaling pathways and, as GSK3 inhibition occurs downstream in the signaling cascade, it bypasses potential opportunities for achieving specificity or regulation at the receptor level. Additionally, the specific roles of individual FZD receptors in anterior-posterior patterning are poorly understood. Here, we have characterized the cell surface expression of FZD receptors in neural progenitor cells with different regional identity. Our data reveal unique upregulation of FZD5 expression in anterior neural progenitors, and this expression is downregulated as cells adopt a posterior fate. This spatial regulation of FZD expression constitutes a previously unreported regulatory mechanism that adjusts the levels of β-catenin signaling along the anterior-posterior axis and possibly contributes to midbrain-hindbrain boundary formation. Stimulation of Wnt/β-catenin signaling in hPSCs, using a tetravalent antibody that selectively triggers FZD5 and LRP6 clustering, leads to midbrain progenitor differentiation and gives rise to functional dopaminergic neurons *in vitro* and *in vivo.*

## Teaser

**Highlighted Article:** A tailored synthetic growth factor that selectively activates the FZD5:LRP6 receptor complex patterns neural progenitor cells into midbrain fate and gives rise to functional dopaminergic neurons *in vitro* and *in vivo*.

## INTRODUCTION

During neural development, after neural induction, the Wnt/β-catenin signaling pathway establishes the anterior-posterior axis by partitioning the nascent central nervous system into forebrain, midbrain, hindbrain and spinal cord (Mulligan and Cheyette, 2012; Nordström et al., 2002). Activation of Wnt/β-catenin signaling leads to posterior patterning of the neural plate while secretion of Wnt antagonists is required for anterior specification. Mutations causing defects in Wnt/β-catenin signaling activity lead to alterations in brain patterning and to truncation of specific brain regions (Lekven et al., 2001; Prakash et al., 2006; Shimizu et al., 2005). The Wnt/β-catenin pathway is a complex network of 19 different Wnt proteins, 10 frizzled (FZD) receptors and several co-receptors (Steinhart and Angers, 2018). Wnt proteins and secreted antagonists of the pathway display both spatial and temporal expression patterns during early embryonic neurodevelopment to tightly regulate regional specification and differentiation into distinct neural subtypes (Brafman and Willert, 2017). However, which of the ten FZD receptors is or are involved during neuropatterning and whether FZD receptors are differentially regulated during this process remain poorly studied. Additional insights into these processes will not only shed light on the role of FZD receptors and Wnt signaling during development and its implications in diseases, but will also provide information about how to more precisely control Wnt signaling during directed differentiation of human pluripotent stem cells (hPSC) to derive more physiologically relevant cell types for regenerative medicine (Chidiac and Angers, 2023; Loh et al., 2016; Mahla, 2016).

The depletion of dopaminergic (DA) neurons in the ventral midbrain is a hallmark of Parkinson's disease (PD), spurring significant efforts to derive these neurons from hPSCs for cell therapy (Barker et al., 2017; Piao et al., 2021; Sonntag et al., 2018). Genetic studies have demonstrated that DA neurons arise from the LMX1A- and FOXA2-expressing mesencephalic floor plate during neural development (Ono et al., 2007). Guided by this knowledge, numerous differentiation protocols have been developed to recapitulate the signaling cues of this embryonic region to pattern hPSC-derived neural progenitor cells (NPCs) into ventral midbrain (VM) fate in order to generate functional DA neurons (Arenas et al., 2015; Doi et al., 2014; Kirkeby et al., 2012; Kriks et al., 2011). In all existing protocols, a small molecule GSK3 inhibitor is employed to activate Wnt signaling, driving the posteriorization of neural progenitors toward a midbrain fate. Notably, midbrain patterning from hPSCs is not efficiently achieved with recombinant Wnt proteins or Wnt-conditioned media (Kriks et al., 2011). This challenge may be attributed to the hydrophobic nature of Wnt ligands due to a post-translational modification that is essential for signaling but complicates their biochemical purification and *in vitro* application (Janda et al., 2012). However, GSK3α/β kinases are pleiotropic kinase also involved in FGF, Hedgehog, Notch, insulin and mTOR signaling, which are pathways known to be implicated in neural progenitor self-renewal and differentiation (Hur and Zhou, 2010; Kim et al., 2011). Furthermore, as inhibition of GSK3 occurs distally in the signaling pathway, it bypasses normal regulatory mechanisms in place to achieve efficient patterning. The recent development of synthetic growth factors that selectively target receptors to activate signaling holds the potential to enhance DA neuron differentiation methods by providing a more precise and controlled activation of Wnt/β-catenin signaling (O'Brien et al., 2023).

Previously, our group has identified selective synthetic antibodies for each individual FZD receptor using phage-display libraries (Pavlovic et al., 2018; Steinhart et al., 2017). Moreover, we have developed modular tetravalent antibodies that can cluster FZD and LRP co-receptors to mimic Wnt ligands (Chidiac et al., 2021; Tao et al., 2019). This FZD-LRP agonist (FLAg) platform can selectively target one or multiple FZD receptors with complete precision to potently activate Wnt/β-catenin signaling. Enabled by these novel tools, we sought to characterize the surface expression of FZD receptors during neural differentiation of hPSCs in order to guide the activation of Wnt signaling rationally through FZD receptors and direct AP patterning of NPCs.

Here, we discover that FZD5 is selectively upregulated in anterior progenitors after neural induction of hPSCs and that its expression is dynamically downregulated as cells adopt a posterior fate. Our work uncovers a previously unreported regulatory mechanism governing the spatiotemporal cell surface expression of a Wnt receptor, thereby forming a feedback regulatory loop dampening the reception of posteriorization signals. Leveraging these findings, we used a FZD5-selective FLAg to pattern neural progenitors into midbrain with high efficiency. The midbrain progenitors derived using this protocol give rise to functional DA neurons *in vitro* and rescue the motor dysfunction when engrafted in the 6-hydroxydopamine (6-OHDA) hemi-lesion rodent model of PD.

## RESULTS

### FZD5 cell surface expression is uniquely upregulated in anterior neural progenitors

To investigate the spatiotemporal expression of FZD receptors during neural AP patterning, we first optimized a differentiation protocol to generate regional-specific NPCs from H1 human embryonic stem cells (hESCs) using established methods (Kirkeby et al., 2012). During neural induction, the anterior-posterior patterning of NPCs is achieved by titrating the dose of GSK3i (CHIR99021) to modulate levels of Wnt/β-catenin signaling during neural induction (Fig. S1A). In the absence of GSK3i, the NPCs defaulted to forebrain fate (marked by *FOXG1* and *OTX2* expression), whereas treatment with 1 µM of CHIR99021 gave rise to midbrain progenitors (marked by *LMX1A* and *OTX2* expression) and treatment with higher dose of CHIR99021 (>2 µM) posteriorized the NPCs to hindbrain fate (*HOXA2* expression) (Fig. S1B). The patterning of the NPCs was validated using immunostaining for FOXG1 and OTX2, and LMX1A and OTX2 (Fig. S1C,D).

**Fig. 1.**
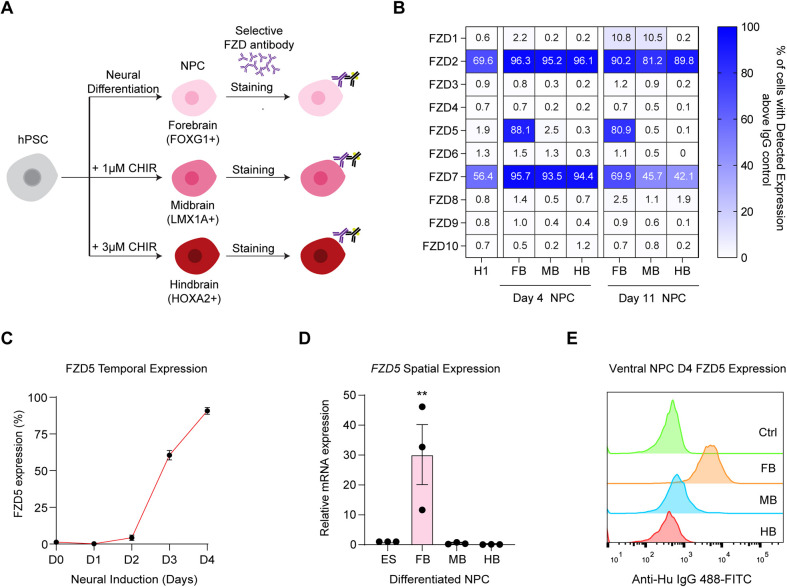
**FZD5 cell surface expression is uniquely upregulated in anterior neural progenitors.** (A) Schematic outlining the derivation of regional neural progenitors from hPSCs and antibody-based profiling of surface expression of FZD receptor. (B) The percentages of expression of each FZD in H1 hESCs, and in dorsal patterned FB (forebrain), MB (midbrain) and HB (hindbrain) NPCs at day 4 and day 11 quantified using flow cytometry. Data are presented as the mean percentage of three replicates. (C) The dynamics of FZD5 surface expression in the first 4 days of neural induction without Wnt stimulation. *n*=3 independent experiments; data are mean±s.e.m. (D) The spatial expression of *FZD5* in patterned regional NPCs at day 11. *n*=3 independent experiments, data are mean±s.e.m. **P<0.01 (two-tailed Student's t-test). (E) The histogram of cell surface expression of FZD5 in ventral patterned regional NPCs at day 4. Data are representative of three independent experiments.

Next, we employed flow cytometry to profile the cell surface expression of each of the ten FZD receptors in regionally patterned NPCs using selective FZD antibodies (Fig. 1A). Our observations revealed that FZD2 and FZD7 exhibit robust expression both in the pluripotent state and in NPCs, regardless of their regional identity (Fig. 1B). In contrast, we identified a unique upregulation of FZD5 in anterior progenitors, with its expression rapidly declining as the cells adopt a posterior identity (Fig. 1B, Fig. S1E). Intriguingly, FZD5 was not expressed in the pluripotent state, but after 4 days of neural induction, this receptor became ubiquitously expressed at the cell membrane of neural-induced cells without posterior patterning (Fig. 1C). Furthermore, the *FZD5* mRNA expression was also uniquely upregulated in the anterior NPCs, indicating that the spatial expression is transcriptionally regulated (Fig. 1D). The spatiotemporal expression pattern of FZD receptors is consistent in both H9 hESCs and WTC11 human induced pluripotent stem cells (hiPSCs) during neural patterning (Fig. S1F). Importantly, FZD5 cell surface expression remained upregulated in anterior neural progenitors after ventral patterning conditions (Fig. 1E), indicating its potential as a target for ventral midbrain patterning using a selective FZD5 FLAg antibody agonist, given its early upregulation during the neural induction phase.

### A genome-wide CRISPR screen identifies regulators of FZD5 expression during neural induction

**Fig. 2.**
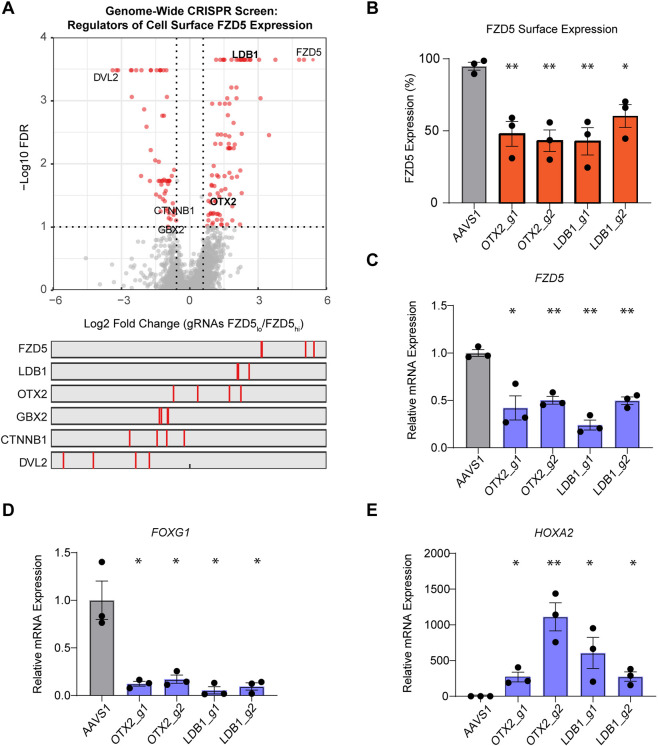
**A genome-wide CRISPR screen identifies regulators of FZD5 expression during neural induction.** (A) Volcano plot depicting the gene level summary of gRNAs that regulate FZD5 surface expression identified in the CRISPR screen. Red dots represent regulators with FDR<0.1. The bottom panel is the individual gRNA enrichment of the highlighted genes. (B) Percentage of FZD5 surface expression in *OTX2*-KO cells and *LDB1*-KO cells at day 4 of neural induction compared with control *AAVS1*-KO cells. Two different gRNAs targeting *OTX2* and *LDB1* were used for validation. *n*=3 independent experiments. Data are mean±s.e.m. A two-tailed Student's *t*-test was used to test for significance. (C) *FZD5* mRNA expression at day 4 of neural induction in *OTX2*-KO cells and *LDB1*-KO cells. *n*=3 independent experiments. Data are mean±s.e.m. A two-tailed Student's *t*-test was used to compare control with *AAVS1*-KO cells. (D,E) *FOXG1* (D) and *HOXA2* (E) mRNA expression at day 4 in *AAVS1*-KO cells compared with *OTX2*-KO cells and *LDB1*-KO cells. *n*=3 independent experiments. Data are mean±s.e.m. A two-tailed Student's *t*-test was used to test for significance. *P<0.05, **P<0.01.

Intrigued with the unique spatial expression of FZD5 in the anterior NPC, we sought to understand the mechanisms governing its expression by conducting a genome-wide CRISPR screen during neural induction (Fig. S2A). The screen was performed in an H1 hESC line harboring doxycycline-inducible Cas9, which was transduced with the Toronto Knockout Library (TKOv3), consisting of 71,090 pooled gRNAs targeting ∼18,000 protein-coding genes (Hart et al., 2017). Subsequently, Cas9 expression was induced before differentiation to generate a population of knockout cells. Next, fluorescence-activated cell sorting was performed to sort the top and bottom 15% of FZD5-expressing cells after 4 days of neural induction. Sorted cells were then processed for quantification of gRNA abundance using next-generation sequencing. The MAGeCK pipeline (Li et al., 2014) was used to determine enrichment of gRNAs in each pool to identify regulators of FZD5 expression (Fig. 2A). Several indicators suggest that the screen performed as expected. First, a higher correlation was observed between the distribution of gRNA within the sorted population and not with the sample replicates (Fig. S2B). Moreover, gRNAs targeting *FZD5* were the most highly enriched in the FZD5-low population (Fig. 2A). Using a false discovery rate (FDR) cut-off of 0.1, we identified 92 positive and 60 negative regulators of FZD5 cell surface levels (Table S1). gRNAs targeting *DVL2* and *CTNNB1*, which are important mediators of Wnt/β-catenin signaling that promote posterior patterning, were expectedly identified in the FZD5-high sorted population. Interestingly, *LDB1* and *OTX2*, which have established roles as forebrain developmental regulators, were uncovered as positive regulators of FZD5 expression (Fig. 2A).

To understand how *LDB1* and *OTX2* regulate FZD5 expression, we selectively knocked out these genes in hESCs using CRISPR-Cas9. We generated two individual polyclonal knockout (KO) lines for each regulator with different gRNAs and quantified KO efficiency using TIDE (Fig. S2C). The KO cells were subjected to neural induction for 4 days and stained for FZD5 expression. The differentiated cells from both *LDB1*-KO and *OTX2*-KO cells had ∼35-50% reduced FZD5 surface expression compared with control cells (Fig. 2B). *FZD5* mRNA expression was also significantly decreased in these cells upon differentiation (Fig. 2C). As OTX2 is known to be important for forebrain and midbrain formation (Millet et al., 1999), we asked whether *OTX2*-KO and *LDB1*-KO cells have altered regional patterning upon neural induction. In the absence of Wnt signaling, NPCs default to forebrain fate, as indicated by a strong induction of *FOXG1* expression on day 11 of differentiation. However, in *OTX2*-KO and *LDB1*-KO, the differentiated NPCs without Wnt activation have significantly reduced *FOXG1* (forebrain marker) and increased *HOXA2* (hindbrain marker) expression (Fig. 2D,E). Reduced FOXG1 expression was also confirmed by immunofluorescence in the *OTX2*-KO and *LDB1*-KO differentiated population (Fig. S2D). Overall, these results elucidate the dynamic regulation of FZD5 cell surface expression along the anterior-posterior axis and indicate that *FZD5* gene expression is dependent on the anterior forebrain regulators OTX2 and LDB1.

### F5L6.13 activates Wnt signaling to pattern VM progenitors from hPSCs

**Fig. 3.**
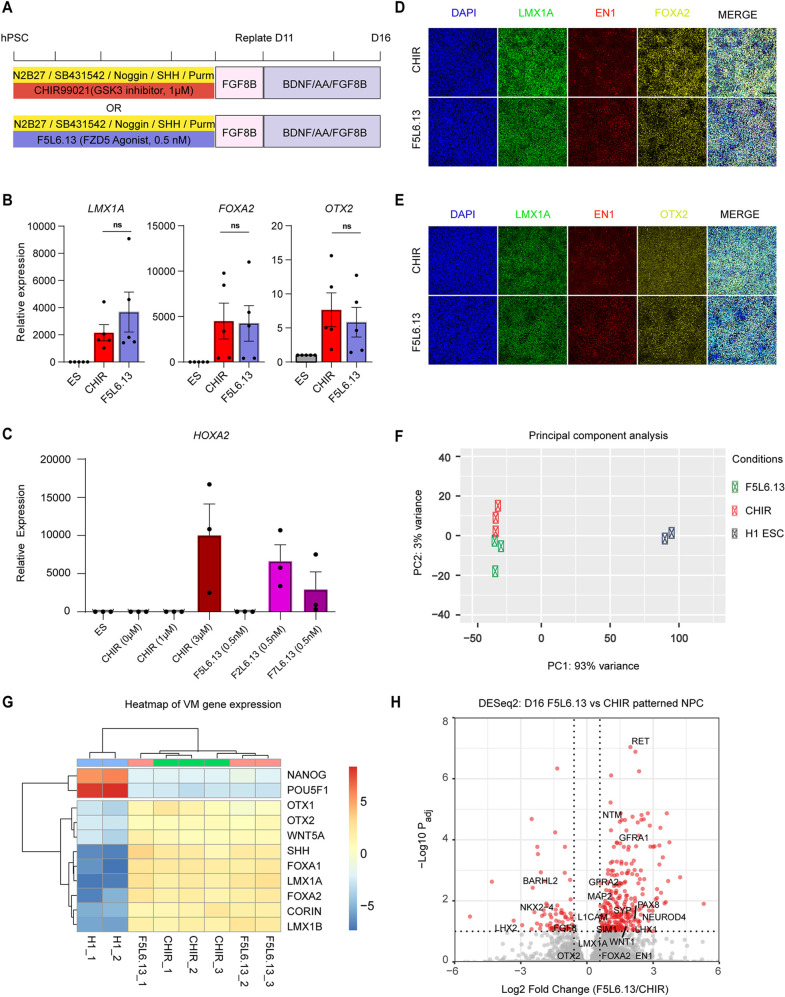
**F5L6.13 activates Wnt signaling to pattern VM progenitors from hPSCs.** (A) Schematic of VM progenitor differentiation protocol from hPSCs. (B) qPCR gene marker expression of *LMX1A*, *FOXA2* and *OTX2* in day 16 differentiated cells patterned with CHIR99021 or F5L6.13, and H1 ESCs (ES). Data are mean±s.e.m. *n*=5 independent experiments. (C) qPCR gene marker expression of hindbrain marker *HOXA2* in differentiated cells patterned with CHIR99021 F5L6.13, F2L6.13 or F7L6.13 at the indicated dose. Data are mean±s.e.m. *n*=3 independent experiments. (D,E) Immunofluorescence staining of VM markers LMX1A, FOXA2 and EN1 (D) and LMX1A, OTX2 and EN1 (E) on day 16. Images are representative of three independent experiments. Scale bar: 100 μm. (F) PCA plot of H1 hESCs and patterned VM progenitors from CHIR99021 or F5L6.13. (G) Heatmap of scaled gene expression of VM markers across treatment conditions. (H) Volcano plot portraying RNA-seq differential expression analysis comparing day 16 patterned VM progenitor. Significantly regulated genes that are associated with midbrain development are highlighted in red with adjusted *P*<0.1 and fold change>1.5.

Based on the FZD profiling data and our finding that FZD5 expression is dynamically regulated during anterior-posterior neural patterning, we hypothesized that selectively activating FZD5 would efficiently induce midbrain patterning. In contrast, activating FZD2 or FZD7, which exhibit constant cell-surface expression, could result in posterior fates due to sustained signaling. To test this, we first performed a dose titration of a selective FZD5 FLAg, F5L6.13, and compared AP patterning of NPCs obtained with CHIR99021 by monitoring expression of various region-specific markers using qPCR (Fig. 3A and Fig. S3A). Strikingly, treatment with 0.5 nM F5L6.13 induced expression of the mesencephalic floor-plate markers *LMX1A*, *OTX2* and *FOXA2* with a similar efficiency to 1 µM CHIR99021, with minimal induction of the posterior marker *HOXA2* (Fig. 3B,C). In contrast, stimulation of the cells with 0.5 nM of F2L6.13 or F7L6.13 to activate the FZD2:LRP6 or the FZD7:LRP6 receptor complexes led to strong induction of *HOXA2* (Fig. 3C). Immunofluorescence staining demonstrated that cell populations derived using F5L6.13 are similar to cells derived using CHIR99021. Both exhibit positivity for LMX1A, FOXA2 and EN1, and for LMX1A, OTX2 and EN1, thereby confirming VM progenitor identity (Fig. 3D,E). In addition, we engineered H1 hESCs to create a LMX1A-GFP fiducial reporter line by inserting an IRES-GFP fragment within the *LMX1A* 3′UTR immediately after the stop codon using CRISPR-Cas9 gene editing methodology (Ran et al., 2013). This line was validated, using regional-specific NPC differentiation conditions, to report only *LMX1A* expression when midbrain patterning was achieved (Fig. S3B). Both CHIR99021 and F5L6.13 treatments led to reporter expression in well over 80% of the cells (Fig. S3C). Moreover, 0.5 nM F5L6.13 patterned VM progenitors efficiently from H9 hESCs and WTC11 iPSCs (Fig. S3D). We conclude that the spatiotemporal regulation of FZD5 cell surface expression during AP patterning provides a previously unreported regulatory mechanism for reinforcing midbrain specification while minimizing hindbrain fate in hPSC differentiation.

Next, we performed bulk RNA-Seq to examine gene expression variations between VM progenitors derived from F5L6.13 and CHIR99021 treatments at day 16. This is the timepoint when progenitor cells can be engrafted in preclinical models of PD to restore motor deficits (Nolbrant et al., 2017). Principal component analysis demonstrated that progenitors patterned from both F5L6.13 and CHIR99021 treatments are overly similar in transcriptomic profile (Fig. 3F). Importantly, examining the top 15 gene loadings contributing to the principal component axis 1 suggested that the pluripotency marker genes *POU5F1*, *TDGF1* (*CRIPTO*) and *ZFP42*, and the midbrain genes *LMX1A*, *FOXA1* and *SHH* are driving the difference between hESCs and differentiated progenitors (Fig. S3E). Furthermore, both treatment conditions led to equivalent increase in VM marker gene expression (Fig. 3G). Focusing on the differences between the VM progenitors, we performed differential gene expression analysis between the two treatment conditions using DESeq2 (Love et al., 2014). The analysis revealed 269 significantly upregulated and 72 significantly downregulated genes in F5L6.13-derived cells when compared with CHIR99021-derived cells (Fig. 3H). Unsurprisingly, given the principal component analysis mentioned above, the canonical VM marker genes *LMX1A*, *FOXA2* and *OTX2* were not among the differentially expressed genes. Interestingly, *PAX8*, *WNT1* and *GFRA1* were upregulated in the F5L6.13 patterned progenitors (Figs 3H and S3F). These genes are highly expressed in the caudal midbrain and correlate with higher DA neuron differentiation efficiency in transplantation studies (Gantner et al., 2020; Kee et al., 2017; Kirkeby et al., 2017). We conclude that F5L6.13 can replace GSK3i for achieving VM patterning during hPSC differentiation and that F5L6.13-patterned progenitors exhibit a more-caudal midbrain fate than CHIR99021-pattened progenitors.

### F5L6.13-patterned VM progenitors give rise to DA neurons *in vitro* and rescue the motor dysfunction of 6-OHDA rat model *in vivo*

**Fig. 4.**
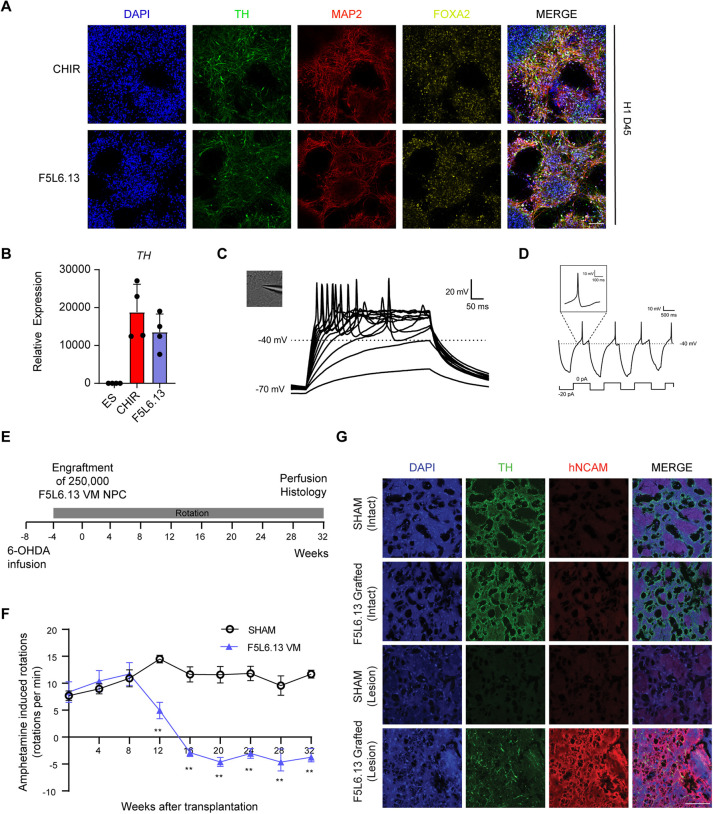
**F5L6.13-patterned VM progenitors give rise to DA neurons *in vitro* and rescue the motor dysfunction in the 6-OHDA rat model *in vivo*.** (A) Immunostaining for TH, MAP2 and FOXA2 in day 45 differentiated neuronal cultures from VM progenitors patterned with CHIR99021 or F5L6.13 in H1 hESCs. Images are representative of three independent experiments. Scale bars: 100 μm. (B) qPCR gene expression of *TH* in day 45 differentiated neuronal cultures normalized to H1 hESCs. Data are mean±s.e.m. *n*=4 independent experiments. (C) Phase-contrast image of a patch-clamped neuron and representative traces of evoked action potentials in F5L6.13 patterned neuronal culture at day 45. Thirteen out of 13 patch-clamped neurons showed traces with action potential. (D) Example of rebound action potentials after brief hyperpolarization in the patch-clamped neurons. (E) Schematic summary of an *in vivo* study with athymic nude rats. (F) Amphetamine-induced rotations of the 6-OHDA lesioned animals in sham-engrafted (*n*=8 rats) or F5L6.13 VM NPC-engrafted (*n*=7 rats) groups. Unpaired multiple *t*-tests were used for comparing engrafted F5L6.13 VM NPCs with sham controls at each timepoint (**P<0.01). (G) Immunostaining of TH and human NCAM in sectioned brain slices of the intact and 6-OHDA lesioned side of the striatum in sham-engrafted and F5L6.13 VM NPC-engrafted animals at 8 months post-transplantation. Scale bar: 100 μm. Images are representative of all the animals in each cohort.

To validate that F5L6.13-patterned VM progenitors can be terminally differentiated into DA neurons, we re-plated the progenitors in a neuronal induction medium to promote neurogenesis. We performed time-course gene expression analysis throughout the differentiation of the VM NPC patterned with CHIR99021 or F5L6.13. The expression of *NR4A2*, a transcription factor that is required for DA neuron development (Zetterström et al., 1997), and *SYP*, a marker of neurogenesis, was not present at D0 and D16 (progenitor stage), but the expression of these genes was induced at D30, and highest expression was observed at D45, indicating the formation of DA neurons (Fig. S4A). Next, using immunostaining, we observed the presence of MAP2^+^ neuron-rich cultures that were also positive for tyrosin hydroxylase (TH) and FOXA2, confirming DA neuron identity, in both CHIR99021 and F5L6.13 treatment conditions at D45 (Fig. 4A). Moreover, LMX1A and OTX2 expression were maintained in these neuronal cultures, indicating midbrain fate (Fig. S4B). In addition, a high induction of *TH* mRNA expression was observed in the differentiated cell populations (Fig. 4B). The upregulation of *SOX6* and *GIRK2* was also observed in the neuronal population, indicating that the A9 DA neuron subtype is present (Fig. S4D). Next, to examine whether the F5L6.13-patterned cell population possess functional characteristics of DA neurons and are excitable *in vitro*, we performed whole-cell patch-clamp electrophysiological recordings in cells exhibiting neuronal morphology with attachments to neuron-like processes at day 45. The neurons showed evoked action potentials upon depolarization and rebound action potentials after brief periods of hyperpolarization, consistent with DA neuron electrophysiological characteristics (Fig. 4C,D). Moreover, the resting membrane potential of the patched neurons in the F5L6.13 condition did not differ from the neurons patterned with CHIR99021 (Fig. S4C).

To investigate whether VM progenitors patterned with F5L6.13 can give rise to functional DA neurons *in vivo*, we employed the 6-OHDA neurotoxin-induced PD rat model that is widely used to test behavioral alterations upon DA depletion (Kirik et al., 1998). The motor dysfunctional phenotype can be rescued upon implantation of cell sources that give rise to functional DA neurons (Kriks et al., 2011; Nolbrant et al., 2017). The athymic nude rats were injected with 6-OHDA unilaterally in the medial forebrain bundle to deplete DA neurons (Fig. 4E). Successful lesions were confirmed with amphetamine-induced rotation 4 weeks later (Fig. 4F). The rats were then separated into two groups and were engrafted with 250,000 F5L6.13-patterned D16 VM progenitors or vehicle-only sham control. We measured the behavioral recovery using amphetamine-induced rotational behavior in animals every 4 weeks until 32 weeks after engraftment. For the animals in the sham control group, the behavioral impairments, as measured by ipsilateral rotations, were present for the duration of the 32 weeks (Fig. 4F). In contrast, the animals receiving VM progenitors showed a significant recovery in amphetamine-induced rotations beginning at week 12 and continually improving and lasting until the 32 weeks endpoint of the experiment (Fig. 4F). This is consistent with previous literature, where the engraftment of VM progenitors requires 12-16 weeks for neuronal maturation to display behavioral benefits (Kriks et al., 2011; Nolbrant et al., 2017). At 32 weeks post-transplantation, the animals were euthanized and histology staining was performed to examine for the presence of human NCAM (a marker of human neuronal cells) and TH in the striatum of the animals (Fig. 4G). In the sham-control group, we saw complete depletion of TH and absence of NCAM staining in the 6-OHDA lesioned side. In the animals engrafted with the VM progenitors patterned with F5L6.13, the presence of NCAM and TH staining was observed, indicating that F5L6.13-patterned progenitors gave rise to human-derived DA neurons. These data suggest that the VM progenitors derived from F5L6.13 treatment give rise to bona fide DA neurons and are functional *in vivo* to efficiently rescue motor dysfunction.

Next, we were curious to determine whether VM progenitors obtained with F5L6.13 were compatible for the generation of three-dimensional organoid models and whether DA neurons would be produced in this context. We adopted a previously established protocol to form midbrain organoids (Jo et al., 2016), wherein, similar to the monolayer differentiation protocol, CHIR99021 is applied during days 0-7 to establish midbrain patterning in embryoid bodies (EBs). The resultant patterned EBs are then encapsulated in Matrigel droplets and grown in a neuronal induction medium under orbital shaking condition. We compared VM organoids patterned with 0.8 µM CHIR99021 or 0.5 nM F5L6.13. Both treatment conditions gave rise to organoids with similar growth rate and gene expression profiles for DA markers *LMX1A*, *OTX2*, *TH*, *SYP* and *MAP2* at day 30 (Fig. S4E,F). In addition, immunofluorescence staining revealed that both conditions yielded TH^+^ cells expressing MAP2, revealing cells with DA neuron identity (Fig. S4G). Moreover, these organoids contain cells with LMX1A and OTX2 expression, indicating midbrain identity (Fig. S4H). These results demonstrate that selective activation of Wnt/β-catenin signaling with F5L6.13 is sufficient to generate midbrain organoids harboring DA neurons.

### scRNA sequencing of day 11 VM progenitors patterned by F5L6.13 and CHIR99021

**Fig. 5.**
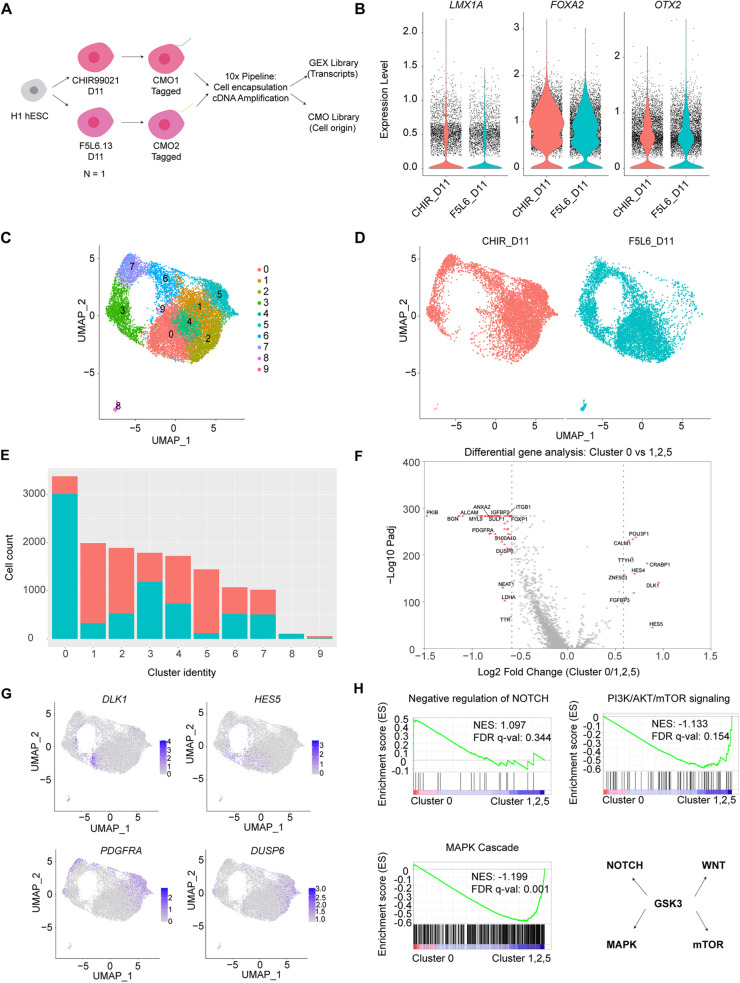
**scRNA sequencing of day 11 VM progenitors patterned by F5L6.13 and CHIR99021.** (A) Schematic of the workflow in 10x Cellplex pipeline labelling the differentiated cells with CMOS for barcoding before sample processing and sequencing. This experiment was performed using one biological replicate in parallel. (B) Violin plots displaying distribution of expression levels of *LMX1A*, *FOXA2* and *OTX2* across all cells. (C) UMAP plot showing clustering of single cells from both treatment conditions. (D) UMAP plot showing the cell identity of treatment conditions contributing the clusters. (E) Bar plot showing the number of cells from each treatment condition contributing to each cluster. (F) Volcano plots of differentially expressed genes comparing cluster 0 versus cluster 1, 2 and 5. Each dot represents a gene. Red dots represent significantly regulated genes with fold change>1.5. (G) Normalized expression levels of indicated genes that were identified in the analysis superimposed onto the UMAPs. Data are colored according to expression levels in each cell. (H) Enrichment of pathways identified using GSEA with a curated ranked gene list from differential expression analysis comparing cluster 0 with clusters 1, 2 and 5. NES and FDR q-values are indicated in the pathway. Bottom right panel indicates the pleiotropic effect of GSK3 in the signaling pathways.

Minimizing cellular heterogeneity during hPSC differentiation is a desired objective in advancing regenerative medicine and transplantation therapies. To investigate the cellular heterogeneity of differentiated VM progenitor populations obtained with F5L6.13 and CHIR99021, and to assess whether VM patterning is successfully achieved by F5L6.13 in comparison with conventional protocols, we conducted single-cell RNA sequencing (scRNA-seq) of cell populations. We employed the 10x CellPlex platform to simultaneously profile the differentiated VM progenitors patterned by F5L6.13 and CHIR99021 at day 11 in parallel within the same batch using one biological replicate (Fig. 5A). The resulting transcriptomic dataset included a total of 14,468 cells across two conditions, with 7433 cells and 7035 cells analyzed in the CHIR99021 and F5L6.13 conditions, respectively. The distribution of transcript counts was similar between the two treatments (Fig. S5A). Both treatment conditions gave rise to a high distribution of cells expressing *LMX1A*, *FOXA2* and *OTX2*, confirming successful VM patterning (Fig. 5B). Moreover, transcripts marking forebrain (*FOXG1*), hindbrain (*HOXA2*) or pluripotent cells (*OCT4*) were not detected in the dataset, indicating the high purity of VM progenitors.

Next, we employed Uniform Manifold Approximation and Projection (UMAP)-based embedding to visualize the clustering of the cell populations. The UMAP segregated the dataset into 10 distinct clusters (Fig. 5C). Interestingly, cells from both treatment conditions contributed to every cluster in the UMAP but with different proportions (Fig. 5D,E, Fig. S5B). We first examined whether our treatment conditions produced cell clusters that corresponded to day 11 cell populations derived from floor plate induction protocols in the literature. We compiled a list of VM progenitor marker genes (Table S2) and then applied the UCell method for scoring the expression of the resulting gene signature in individual cells (Andreatta and Carmona, 2021). Most cells from both treatment conditions expressed the VM progenitor gene signature (Fig. S5C). In addition, we compared our dataset with another scRNA dataset from day 11 VM progenitor patterned with CHIR99021 that identified three separate clusters of floor-plate progenitors (Nilsson et al., 2021). We extracted the gene signatures associated with each of the three floor-plate clusters and applied UCell scoring to our dataset (Table S2). High expression scores for each of the three floor plate gene signatures was observed in our dataset, highlighting the efficiency of VM patterning in our differentiation protocol (Fig. S5D). Moreover, the obtained cell populations expressed high levels of *NES* and *SOX2* (neural progenitor markers), with minimal *ASCL1* (a neurogenesis marker) and *SNAP25* (a neuronal marker) expression, indicating that the day 11 cells from the dataset were in the neural progenitor stage (Fig. S5E).

To identify the differences between CHIR99021- and F5L6.13-patterned VM progenitors, we focused on the clusters that showed distinct enrichment in each treatment (Fig. 5E, Fig. S5B)*.* Specifically, CHIR99021-patterned cells were over-represented in clusters 1, 2 and 5 but under-represented in cluster 0 when compared with F5L6.13-patterned cells*.* The canonical VM markers *LMX1A*, *FOXA2* and *OTX2* were consistently expressed across these clusters, indicating that these differences did not arise from improper VM patterning (Fig. S5F). To examine whether the observed differences could be attributed to differences in cell cycle status, we employed the CellCycleScoring function in Seurat to classify cell cycle phases in the population. The UMAP with cell cycle classification revealed that clusters 6 and 7 are associated with cells in G2M phase, while cluster 3 includes cells in S phase (Fig. S5G). Moreover, a similar proportion of cells in each cell cycle phase was observed across the two treatments, suggesting that the differences in cluster abundance is not merely due to varying cell cycle status. Next, we performed differential gene expression analysis comparing clusters 0 with cluster 1, 2 and 5, and identified 11 significantly upregulated genes and 57 downregulated genes with a fold change greater than 1.5 (Fig. 5F). Interestingly, the analysis revealed that the Notch pathway target genes *DLK1* and *HES5* are upregulated in cluster 0. Conversely, *DUSP6* and *PDGFRA*, which is involved in the MAPK cascade, were found to be upregulated in clusters 1, 2 and 5 (Fig. 5G). This prompted us to investigate differences more broadly at the pathway level in CHIR99021- and F5L6.13-patterned cells, with an emphasis on pathways associated with GSK3 function. Thus, we curated a ranked gene list from the cluster comparison and performed GSEA. Interestingly, differential enrichment of Notch, MAPK and mTOR pathways were observed. Consistent with the results above, F5L6.13-patterned progenitors exhibited an increase in the proportion of cell clusters exhibiting modulation of Notch signaling while CHIR99021-patterned progenitors gave rise to an increased proportion of clusters with modulation of MAPK and mTOR pathways (Fig. 5H). This suggests that individual cells derived from the two treatments show differential regulation of several signaling pathways known to involve GSK3. Overall, our single-cell transcriptome profiling of day 11 cell populations shows that using CHIR99021- and F5L6.13-patterned NPC gives rise to VM fate with high purity.

### scRNA sequencing of day 30 VM neuronal population patterned by F5L6.13 and CHIR99021

**Fig. 6.**
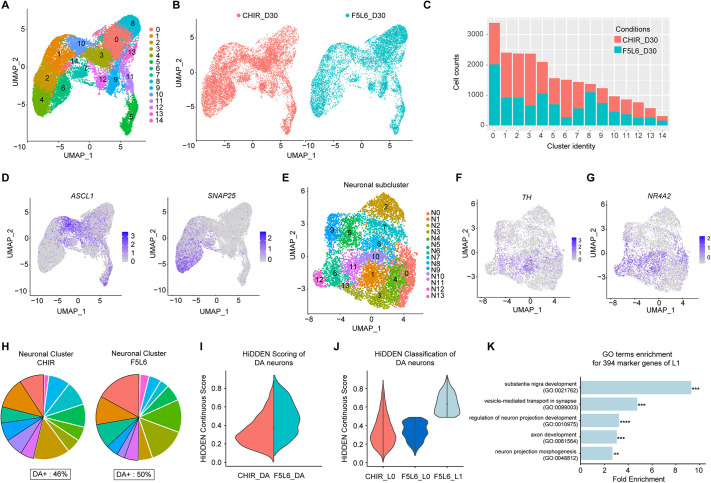
**scRNA sequencing of day 30 VM neuronal population patterned by F5L6.13 and CHIR99021.** (A) UMAP plot showing clustering of the day 30 single cells from one biological replicate. (B) UMAP plot showing the cell identity of treatment conditions (CHIR99021 or F5L6.13) contributing the clusters. (C) Bar plot showing the number of cells from each treatment condition contributing to each cluster. (D) Normalized expression levels of *ASCL1* and *SNAP25* superimposed onto the UMAPs. Data are colored according to scaled expression in each cell. (E) UMAP plot showing subclustered neuronal population. (F,G) Normalized expression levels of *TH* (F) and *NR4A2* (G) superimposed onto the neuronal subclustered UMAP. Data are colored according to expression levels in each cell. (H) Pie chart displaying the proportion of neuronal subclusters contributed from each treatment condition with the DA^+^ clusters highlighted. The percentage of DA^+^ cluster in each condition is indicated. (I) HiDDEN continuous scoring of the DA neuronal population in CHIR and F5L6.13 conditions. (J) Classification of the DA subpopulation from the continuous score generated from HiDDEN. (K) Gene ontology enrichment analysis of the 394 marker genes of the F5L6_L1 subpopulation. **P<0.01, ***P<0.001, ****P<0.0001 (Fisher's Exact test).

To evaluate whether the different VM progenitor cell clusters observed at day 11 in the CHIR99021 and F5L6.13 treatment conditions lead to distinct neuronal populations, we continued the differentiation protocol to day 30 and performed scRNA-seq using 10x CellPlex on the resulting cell populations using one biological replicate. The resulting transcriptomic analysis included 12,757 cells in the CHIR99021 condition and 10,468 cells for F5L6.13, with similar distribution of transcript and gene counts (Fig. S6A). UMAP-based embedding partitioned the cell populations into 15 clusters (Fig. 6A). Similar to the day 11 clusters, cells from both treatment conditions contributed to every cluster in the UMAP (Fig. 6B,C), and a high proportion of cells expressing *LMX1A*, *FOXA2* and *OTX2* were found in both treatments, indicating the cell population is indeed of VM fate (Fig. S6B). To examine cell cycle status, we again used the cell-cycle related gene set to calculate and assign cell-cycle scores to each cell. This revealed that both treatments gave rise to cells with a similar proportion of cell-cycle states and, as expected for this stage, a trend reflecting the progression from proliferating progenitors to postmitotic neuronal cell population (Fig. S6C).

To identify the clusters representing the neuronal populations, we assessed the expression of neural progenitor markers *NES* and *SOX2*, neurogenesis marker *ASCL1*, and neuronal marker *SNAP25* (Fig. 6D, Fig. S6D). Clusters 1, 2, 4 and 6 displayed pronounced neuronal gene expression while demonstrating minimal expression of genes related to progenitor cells. Interestingly, 40.8% and 30.4% of the analyzed cells were attributed to the neuronal clusters when VM progenitors were patterned with GSK3i and F5L6.13, respectively. This suggests that, overall, treatment with GSK3i may be more effective at promoting neuronal differentiation. We then refined the analysis and subjected the neuronal clusters to another UMAP embedding to examine heterogeneity, focusing on the neuronal populations. The subclustered neuronal population parsed into 14 distinct neuronal clusters (N0-N13) (Fig. 6E, Fig. S6E). Feature plots of *TH* and *NR4A2* display that some of the neuronal clusters have DA neuronal identity (Fig. 5F,G). To define the clusters that are DA neurons of VM origin, we classified DA^+^ clusters based on the expression of *TH*, *NR4A2*, *LMX1A* and *FOXA2*. Based on the violin plots of gene expression of these markers, we classified that N0, N1, N6, N9, N10 and N11 clusters are DA^+^ (Fig. S6F). In F5L6.13-patterned cultures, the DA^+^ clusters represent 50% of the neuronal population, while CHIR99021-patterned neuronal cultures exhibit 46% DA^+^ clusters (Fig. 6H). We also conducted immunofluorescence staining of the D30 neuronal culture, revealing comparable TH^+^ populations and similar proportions of NR4A2^+^/TH^+^ and FOXA2^+^/TH^+^ cells in both conditions (Fig. S6H). Based on these results, we conclude that F5L6.13 can achieve similar efficiency to GSK3i in DA neuron differentiation.

To further investigate the transcriptional differences between DA neurons generated from CHIR and F5L6.13-patterned cells, we deployed HiDDEN, a computational method designed towards identifying unique subpopulations of cells in cross-condition experiments (Goeva et al., 2023 preprint). The continuous scores produced by HiDDEN can be interpreted as the per-cell degree of dissimilarity from cells of the control condition, in this case the CHIR DA neurons. The distributions of the continuous scores revealed a difference in the gene expression profiles in the DA neurons generated from the two conditions (Fig. 5I). Subsequently, we employed HiDDEN to cluster the continuous scores of F5L6.13-derived DA neurons into two groups. Cells with lower scores, resembling CHIR-induced cells, were labeled as F5L6_L0, whereas cells with significantly higher scores indicating substantial differences were labeled as F5L6_L1 (Fig. 5J). To validate the lack of transcriptional differences between CHIR_L0 and F5L6_L0 DA neurons, we performed differential expression (DE) analysis resulting in one DE gene (Fig. S6I), suggesting that the F5L6.13 agonist produces a DA neuronal subpopulation that is very similar to the current protocol using GSK3i. However, the F5L6_L1 population significantly differs from CHIR and CHIR-like F5L6_L0 cells, as evidenced by 597 significant DE genes (Fig. S6I, DE genes are listed in Table S3). Gene ontology (GO) enrichment analysis of the 394 DE genes enriched in F5L6_L1 DA neurons revealed that this subpopulation is enriched in GO terms of ‘substantia nigra development’ and ‘neuronal differentiation processes’ (Fig. 5K, Fig. S6J). This analysis suggests that a subpopulation of DA neurons generated by F5L6.13 aligns more closely with the anatomical origin of DA neurons. Overall, F5L6.13 is as efficient as CHIR99021 in directing neural progenitors toward the DA neuron fate.

## DISCUSSION

Successful PSC differentiation into functional cell types has been guided by knowledge of the endogenous developmental signals regulating differentiation of progenitor cells into tissue-specific cell types. Wnt proteins are one such important class of evolutionarily conserved signaling molecules, activating β-catenin signaling to mediate embryonic and tissue stem cell self-renewal and differentiation (Steinhart and Angers, 2018). During neural development, the role of Wnt/β-catenin signaling for the posteriorization of the AP axis is well established. However, the functional role of individual FZD receptors, as well as their spatiotemporal expression and regulation during this process remains elusive. Although some data exist describing the spatiotemporal mRNA expression of FZD receptors during mouse nervous system development (Fischer et al., 2007), the lack of highly selective antibodies for the ten FZD receptors has hindered examination of their context-specific cell expression. In the present study, we leveraged existing hPSC differentiation methods to generate region-specific NPCs and, using a panel of selective FZD antibodies, discovered that FZD5 is selectively upregulated at the cell surface of anterior NPC (Fig. 1). This is consistent with previous RNA *in situ* hybridization staining showing that *Fzd5* expression is restricted to the forebrain during early mouse development (Fischer et al., 2007) and suggests that FZD5 may be the functionally relevant Wnt receptor implicated in Wnt/β-catenin signaling during AP patterning in humans.

Elucidating the mechanism underlying the spatiotemporal expression of Wnt proteins and their receptors is crucial for better understanding their individual roles during development and tissue homeostasis. In this study using a phenotypic genetic screen in hPSCs, we uncovered that FZD5 expression is governed in part by *OTX2* and *LDB1* (Fig. 2). Previous studies have shown that *OTX2* and *LDB1* are required for anterior neural tube patterning, as knockout of either regulator leads to truncation of anterior head structures in mice (Kinare et al., 2019; Kurokawa et al., 2004). Furthermore, LDB1 and OTX2 function in the same protein complex that is crucial for the formation of the anterior visceral endoderm, a patterning center that is essential for forebrain formation (Costello et al., 2015). Interestingly, in that study, *Fzd5* and *Fzd8* were identified as target genes for LHX1, which is known to function together with OTX2 and LDB1. Consistent with these findings, we found that knockout of *LDB1* and *OTX2* leads to significant decrease in FZD5 expression upon neural induction. Neural specification in these knockout cells is unaffected but the regional identity of the neural progenitors was altered. An important function for OTX2 is to establish the midbrain-hindbrain boundary. In this context, OTX2 expression is opposed by the caudally expressed GBX2 transcription factor to delineate and position the boundary. *Gbx2* knockout mice therefore have an expanded *Otx2* expression domain and exhibit midbrain defects (Millet et al., 1999). Our phenotypic screen further revealed GBX2 as a negative regulator of FZD5 expression (Fig. 2A). The precise control of the spatial expression of FZD5 along the anterior-posterior axis during development, which is regulated by OTX2, may constitute a feedback regulatory loop that reinforces brain regional specification. Within this loop, when progenitors adopt a posterior identity in response to Wnt signaling, the expression of FZD5 is downregulated to limit further reception of posteriorization signals. Supporting this idea, when neural progenitors are stimulated with a FZD2 (F2L6.13) or a FZD7 agonist (F7L6.13) with the same concentration as the FZD5 agonist, the progenitors adopt a more posterior fate, possibly due to the lack of downregulation of cell surface FZD2 and FZD7 during the patterning and to ensuing sustained activation of β-catenin signaling that is required for hindbrain specification (Fig. 3C).

The use of small molecule GSK3i is common in hPSC-directed differentiation protocols when manipulating Wnt/β-catenin signaling is necessary (Kirkeby et al., 2012; Loh et al., 2016). Indeed, Wnt ligands are highly hydrophobic glycoproteins, and their biochemical purification and *in vitro* application are complicated by a post-translational modification that is essential for signaling (Janda et al., 2012). However, several limitations need to be considered when using this indirect means to activate signaling. First, as mentioned above, GSK3 is a kinase involved in multiple signaling pathways, such as FGF, Hedgehog, Notch, insulin and mTOR, that have known roles in progenitor self-renewal and differentiation (Hur and Zhou, 2010). Inhibiting this kinase may therefore cause pleiotropic effects, leading to heterogeneity and decreased functionality of the differentiated population. Second, the dynamics and kinetics of nuclear β-catenin accumulation and localization differ when cells are stimulated with Wnt proteins when compared with GSK3i (Kafri et al., 2016). For example, hESCs respond adaptively to Wnt proteins, whereas they exhibit sustained β-catenin activation in response to GSK3i (Massey et al., 2019). This may be especially important, as integration of signaling dynamics is proposed to be an important feature, along with time and concentration, for patterning of tissues in response to a morphogen (Metzis et al., 2018). Third, treatment with GSK3i activates β-catenin signaling in every cell within a population, thereby limiting the ability to selectively activate the pathway in desired subpopulations expressing a given Wnt receptor. Finally, the deployment of differentiated hPSCs for cell therapy applications requires the biomanufacturing of cell products, which can potentially carry over trace amounts of long-lived small molecules, such as CHIR99021, and cause undesired effects. The short half-life of F5L6.13 protein combined with its increased potency, when compared with GSK3i, could therefore represent an important biomanufacturing advantage.

The recent development of Wnt surrogate molecules now enables the selective and potent activation of FZD-LRP5/6 receptor complexes. Given the limitations described above that are associated with the use of GSK3i, selective FZD agonists may provide significant advantages for directed differentiation of hPSCs. Supporting this, we have previously demonstrated that treatment of hESCs with the pan-FZD FLAg (FPL6.13) led to robust mesoderm induction to levels comparable with GSK3i treatment (Tao et al., 2019). Subsequently, it was demonstrated that F7L6, a FZD7-LRP6 selective Wnt surrogate molecule, promotes mesendoderm differentiation of hESC (Gumber et al., 2020). Interestingly, RNAseq analysis revealed significant differences in gene expression levels and kinetics when hESCs were treated with a single continuous dose of GSK3i compared with F7L6. In the most extreme case, some mesendodermal genes, such as *FOXA2* and *SOX17*, were only induced by F7L6 (Gumber et al., 2020). How these differences influence differentiation outcomes and generation of specific cell types is unknown. We demonstrate the effective use of selective targeting of FZD5 by using the agonist F5L6.13 to activate Wnt signaling. This approach efficiently achieves VM patterning from hPSCs and results in the generation of functional DA neurons (Figs 3 and 4). Moreover, single-cell RNA analysis revealed differential activation of GSK3-mediated signaling pathways when comparing CHIR99021 and F5L6.13-patterned VM progenitors (Fig. 5). Further investigation is warranted to elucidate the specific impacts of these pathway differences on the differentiated cells. Additionally, we observed that a subset of DA neurons generated from F5L6.13-patterned cells exhibits a gene signature closely aligned with the anatomical origin of DA neurons, representing a potentially more desirable neuronal phenotype (Fig. 6). It is important to note that these observations are based on scRNA analysis on one biological replicate and further replicates are required for more robust confirmation.

In conclusion, in this study we have uncovered a previously unreported mechanism regulating the spatiotemporal expression of a Wnt receptor (FZD5) during anterior posterior patterning. Leveraging the selective activation of this receptor and the tight control of Wnt/β-catenin signaling provided by this regulatory mechanism, we developed a novel *in vitro* hPSC directed differentiation protocol that yields functional DA neurons able to efficiently rescue locomotor impairment when transplanted in a rodent model of PD. Our protocol introduces the use of a tailored synthetic growth factor that selectively activates the FZD5:LRP6 receptor complex and faithfully patterns NPCs into midbrain identity, replacing the conventional use of GSK3i. We anticipate that gaining a deeper understanding of the underlying genetic regulatory mechanisms governing Wnt/β-catenin signaling and other signaling pathways, combined with the development of synthetic surrogate growth factors that faithfully mimic endogenous cell-cell communication mechanisms, will play a pivotal role in unlocking the potential of regenerative medicine.

## MATERIALS AND METHODS

### hPSCs culture

The hPSC lines H1 hESC and H9 hESC were obtained from the Wicell Research Institute. WTC11 hiPSC was obtained from the Conklin Lab at Gladstone, UCSF. The H1-iCas9 cells were generated from the H1 hESC parental line Centre for Commercialization of Regenerative Medicine (Toronto, Canada) by insertion of a vector sequence containing the inducible TRE3G-Cas9-P2A-BFP2 and constitutive Tet-On 3G expression cassettes into the safe harbor locus *AAVS1* using CRISPR. The hPSCs were maintained under feeder-free conditions in six-well plates coated with Geltrex (ThermoFisher) in StemFlex media (Gibco) at 37°C in a humidified CO_2_ incubator. The media was changed every other day. Cells were passaged once they reached ∼70-90% confluency via clump passaging with Versene (ThermoFisher). hPSC lines were tested routinely for mycoplasma contamination using the MycoAlert Plus detection kit (Lonza).

### Human NPC Differentiation

Neural differentiation from hPSCs was performed based on previously established neural induction protocol (Kirkeby et al., 2012; Nolbrant et al., 2017). The hPSCs were plated and grown on Geltrex-coated plates in neural induction media (NIM) containing 1:1 DMEM/F12:Neurobasal medium (ThermoFisher), 1:100 N2 supplement (Invitrogen), 1:50 B27 without vitamin A (Invitrogen) and 1% GlutaMAX (Gibco). Ventral NPC patterning was achieved by addition of 10 μM ROCK inhibitor Y27632 (R&D Systems), 10 μM SB431542 (R&D Systems), 100 ng/ml Noggin (Peprotech), 100 ng/ml SHH-C24II (R&D Systems) and 0.5 μM Purmorphamine at day 0 to day 9. Posterior patterning was achieved with CHIR99021 (Sigma-Aldrich) or F5L6.13 (in house) at indicated concentration from day 0 to day 9. The medium was changed on days 2, 4 and 7 with the morphogens without ROCK inhibitor. For VM progenitor differentiation, 100 ng/ml FGF8b (R&D Systems) was added with the neural induction media on day 9 to day 11. On day 11, VM progenitors were dissociated using Accutase (Gibco) and replated on Geltrex-coated dishes in 20 ng/ml BDNF (R&D systems), 0.2 mM ascorbic acid (Sigma Aldrich), 100 ng/ml FGF8b and 10 μM Y-27632. Media were changed on day 14 without ROCK inhibitor. On day 16, the cells were either taken for analysis or re-plated for neuronal differentiation. For neuronal differentiation, the cells were dissociated again with Accutase, and re-plated and grown in neuronal induction medium consisting of Neurobasal, 1:50 B27 without vitamin A and 1% GlutaMAX (Gibco), with 10 μM DAPT (Sigma-Aldrich), 0.2 mM ascorbic acid, 20 ng/ml BDNF, 20 ng/ml GDNF (R&D Systems), 0.5 mM db-cAMP (Sigma-Aldrich) and 10 μM Y-27632 on poly-L-ornithine (Sigma-Aldrich) and laminin (Sigma-Aldrich) coated dishes. All the media were changed using fresh medium every 2-3 days without Y-27632 until the experimental endpoint, as indicated. After day 25, only half of the medium was changed in order to prevent cell detachment.

### Midbrain organoid differentiation

Midbrain organoids were generated as previously described (Jo et al., 2016). Briefly, the H9 ESC were dissociated to single cells and 10,000 cells were plated in ultra-low attachment U-bottom 96-well plates (Corning) to form EBs in NIM containing 10 μM Y-27632, 10 μM SB431542 and 200 ng/ml noggin, and patterned with either 0.8 μM CHIR99021 or 0.5 nM F5L6.13. The medium was changed on day 2 without rock inhibitor. On day 4, the EBs were cultured with the addition of 100 ng/ml SHH-C24II and 100 ng/ml FGF8b in the cocktail. On day 7, the media was completely removed and 30 μl of ice-cold reduced-growth factor Matrigel (Corning) was immediately added to each well using a pre-chilled pipette tip. The Matrigel-embedded midbrain organoid was placed into a 37°C incubator for 30 min to allow the Matrigel to solidify, and was grown in tissue growth induction medium containing Neurobasal medium, 1:100 N2 supplement (Invitrogen), 1:50 B27 without vitamin A (Invitrogen), 1% GlutaMAX (Invitrogen), 1% minimum essential media-nonessential amino acid (Invitrogen) and 0.1% β-mercaptoethanol (Invitrogen) supplemented with 2.5 μg/ml insulin (Sigma-Aldrich), 200 ng/ml laminin (Sigma-Aldrich), 100 ng/ml SHH-C24II and 100 ng/ml FGF8. To promote growth and differentiation, the organoids were transferred into ultra-low-attachment six-well plates (Corning) using a cut 1000 μl pipette tip the following day. The organoids were then grown in the differentiation media, which consisted of Neurobasal medium, 1:100 N2 supplement, 1:50 B27 without vitamin A, 1% GlutaMAX, 1% minimum essential media-nonessential amino acid (Invitrogen), 0.1% β-mercaptoethanol (Invitrogen), 10 ng/ml BDNF, 10 ng/ml GDNF, 100 μM ascorbic acid and 125 μM db-cAMP to promote neuronal differentiation under an orbital shaker (ThermoFisher) at 70 rpm. The medium was replaced every 3 days until the indicated time point.

### CRISPR screening

Lentivirus containing the TKOv3 gRNA libraries was prepared as previously described (Hart et al., 2017). Around 100 million H1 iCas9 cells were infected with TKOv3 virus at a multiplicity of infection of 0.3 to aim for library coverage of 400-fold and seeded into Geltrex-coated plates. Next day, the cells were subjected to 1 μg/ml puromycin (Sigma-Aldrich) selection in StemFlex media for 2 days. Next, cells were treated with doxycycline (1.5 μg/ml) for 4 days to induce Cas9 expression and gene editing. After dox selection, cells were split into three biological replicates and were seeded in NIM with 10 μM SB431542, 100 nM LDN-193189 (Sigma-Aldrich) and 10 μM Y-27632. Media was changed on day 2 without Y-27632. Cells were harvested and stained using FZD5 IgG antibody after the flow cytometry staining protocol. After staining, the cells were fixed with 2% PFA for 20 min, then resuspended in sorting buffer at 4°C containing PBS with 5 mM EDTA (Gibco), 25 mM HEPES (Gibco) and 1% BSA. Cells were sorted for the top 15% and bottom 15% of FITC-expressing cells per replicate on a BD Influx and BD FACS Aria III (BD Biosciences). Genomic DNA was extracted from these cells following the protocol of Chen et al. (2015), amplified and barcoded with Illumina TruSeq adapters. Samples were sent for next-generation sequencing at the Lunenfeld-Tanenbaum Research Institute Sequencing Facility (Toronto). Analysis of screen sequences was performed following the MAGeCK pipeline (Li et al., 2014). The FZD5-high expressing cells were compared with FZD5-low expressing cells using the ‘mageck test’ function following default parameters that generated a ranked list of hit gRNAs indicating regulators of FZD5.

### Quantitative PCR

Cells were harvested, pelleted and resuspended in TRIzol Reagent (Life Technologies), and stored at −80°C until extraction. RNA was extracted according to the manufacturer's protocol and quantified using Nanodrop (ThermoFisher). A total of 2 μg of RNA was treated with DNAseI and subsequently cDNA was obtained using SuperScript II Reverse Transcriptase Kit (ThermoFisher). qPCR was performed using 12.5 ng of RNA, the respective primer mix (see qPCR primer list, Table S4) and Power SYBR Green according to manufacturer's protocol (ThermoFisher) in triplicates. Samples were run and analyzed on a CFX384 Real-Time PCR Detection System (BioRad) using the following thermocycling conditions: 95°C for 10 min, followed by 40 cycles of 95°C for 15 s, 60°C for 1 min and 72°C for 30 s. Mean relative gene expression was analyzed with the ΔΔCt method using CFX Maestro software and normalized to the housekeeping gene *PPIB*.

### CRISPR-Cas9 gene editing

For single gene knockout studies, individual gRNAs (see Table S5 for gRNA sequence list) were ligated into Esp3I digested LentiCRISPRv2, a generous gift from Feng Zheng (Addgene, 52961), by following established protocol (Ran et al., 2013). H1 hESCs were infected with the concentrated lentivirus with the gRNA in the LentiCRISPRv2 construct and underwent puromycin selection for 2 days before analysis for knockout efficiency by Tracking of Indels by DEcomposition (TIDE). TIDE analysis was performed on gRNA targeted regions of genomic DNA extracted using the PureLink Genomic DNA Mini Kit and amplified by PCR using KAPA HiFi HotStart ReadyMix PCR (Roche). Samples were Sanger sequenced using respective forward TIDE primers. For the list of primers used for PCR and TIDE analysis, see Table S5. INDELs and sgRNA cutting efficiency were measured by comparing .ab1 files of targeted amplified genomic regions of the wild-type cells with the knockout cells using the online TIDE software (Brinkman et al., 2014).

For H1 LMX1A-GFP generation, the original reporter backbone pJ151-HDR, a generous gift from Alex Kentsis (Addgene, 113631), was used. Synthetic design of pJ151-LMX1A-IRES-EGFP was planned using SnapGene cloning software. Briefly, a IRES-GFP fragment was subcloned into the pJ151 vector downstream of the HDR sites using Gibson Assembly (New England Biolabs). Left and right homology arms (LHA and RHA) from genomic DNA of H1 hESCs were amplified using PCR (Kapa Biosystems), designed for integration immediately after the stop exon of the human *LMX1A* gene (sequence accessed by GenBank file from NCBI). The homology arms were cloned into the backbone using restriction cloning. The LHA was cleaved using enzymes AscI and BAMHI (New England Biolabs), and the RHA was cleaved using SpeI and ClaI (New England Biolabs), and subcloned into the pJ151-HDR-IRES-GFP vector. The CRISPR nickase strategy was used for targeted insertion (Ran et al., 2013). A pair of nickase gRNAs targeting the genomic region near the stop codon of LMX1A was cloned into pX335, a gift from Feng Zhang (Addgene, 42335). For H1 LMX1A-GFP generation, the pair of nickases were electroporated into H1 hESCs with the pJ151-LMX1A-IRES-GFP vector using the Neon Transfection System (Invitrogen) following default parameters. The cells were selected using puromycin and tested for LMX1A-GFP activity. See Table S5 for the list of sequences for primers and gRNA used for H1 LMX1A-GFP generation.

### gRNA lentivirus production

HEK 293T cells were cultured in DMEM (Gibco) with 10% fetal bovine serum (Gibco) and were transfected at ∼60% confluency with 5 μg of a psPAX2 packaging plasmid and 2 μg of a VSV. G enveloping plasmid and 5 μg of the intended LentiCRISPRv2 gRNA construct in 250 μl of OptiMEM (Gibco) reduced serum media. A 3:1 ratio of polyethylenimine (Sigma-Aldrich) to DNA was prepared separately, diluted in OptiMEM and briefly vortexed and incubated for 20 min, then added dropwise to cell culture plates. Media were changed the following day, and 24 h later those media were harvested and spun down (2000 ***g*** for 2 min). Supernatant was filtered with a 0.45 μm filter and Lenti-X Concentrator (Takara) was added to media in a 1:3 volume ratio. Tubes were rotated for 1 h at 4°C then spun at 1500 ***g*** for 45 min at 4°C. The viral pellets were resuspended in 250 μl of DMEM/F12, aliquoted and kept at −80°C for long-term storage.

### Immunofluorescence microscopy

For the monolayer differentiation protocols, the cells were seeded and differentiated on Geltrex-coated round glass cover slips (Fisher Sci). At the timepoint stated, the cells were fixed with 4% paraformaldehyde (PFA) and stored at 4°C until ready for staining. For midbrain organoids, the organoids were fixed in 4% PFA overnight and washed extensively the next day before placing in a 30% sucrose (BioShop) solution in PBS at 4°C overnight, Subsequently, the organoids were then embedded in OCT compound (VWR) for cryosectioning. The frozen organoids were sectioned at 16 μm using a cryostat (Leica) and mounted on Superfrost plus microscope slides (ThermoFisher Scientific). For staining, the slides were permeabilized and blocked in PBS with 0.1% Triton X-100 (Sigma-Aldrich) and 5% donkey serum (Sigma-Aldrich) for 1 h at room temperature. Primary antibody was prepared in blocking solution and added to coverslips for overnight incubation at 4°C, followed by extensive washing. Secondary antibody was added in blocking solution and incubated for 1 h in the dark at room temperature with the cover slips. After washing, the coverslips were mounted onto slides (VWR) using Fluoromount mounting media (Sigma-Aldrich) with DAPI (Sigma-Aldrich). For rat brain imaging, rats were anesthetized with 5% isoflurane before perfusion and then intracardially perfused with 50 ml phosphate-buffered saline (PBS), followed by 50 ml 4% paraformaldehyde in PBS. The brains were removed and stored in 4% paraformaldehyde in PBS overnight at 4°C and then transferred to a 30% sucrose solution for cryoprotection for 48 h at 4°C. Tissue was embedded in the freezing media HistoPrep (Fisher Chemical) and cut in 40 µm coronal sections. Sections were incubated in permeabilizing solution containing 1.2% Triton-X 100 in PBS followed by a blocking solution containing 10% normal goat serum (Thermofisher) in PBS for 1 h to avoid non-specific binding. Sections were then incubated in primary antibody overnight and then briefly washed the next day then incubated secondary antibody in 2% NGS for 1 h and washed. Sections were mounted in Vectashield medium (Vector Laboratories). For the antibody list, see Table S6. All slides were then imaged on a Zeiss LSM 710 confocal microscope. Processing of images was performed with ImageJ.

### Flow cytometry

The cells were harvested using TrypLE (Gibco) for single cell dissociation before staining. For the H1 hESC LMX1A-GFP reporter line, the cells were incubated with the viability dye eFluor 780 (Invitrogen) for 30 min and washed extensively before analysis. For FZD antibody staining, the differentiated cells were harvested and blocked for 1 h with 3% BSA on ice. The cells were then stained with the selective FZD IgG antibody (Pavlovic et al., 2018; Steinhart et al., 2017) for 1 h on ice in the blocking solution. Cells were washed with PBS and stained with goat anti-Human IgG Fc DyLight 488 (Invitrogen) and viability dye eFluor 780 in blocking solution for 30 min at 4°C in the dark. After extensive washing, cells were analyzed by flow cytometry using a Beckman Coulter Cytoflex S instrument (Beckman Coulter). Post-sample analysis was performed in FlowJo v10.

### FZD antibody production

Selective FZD-binding antibody was cloned and produced as previously described (Pavlovic et al., 2018). The Wnt receptor-binding paratopes that comprise F5L6.13 were identified from recombinant selections on human LRP6 and human FZD5 using a phage displayed synthetic Fab library and produced in the tetravalent antibody format using previously described methods (Tao et al., 2019). Binding specificity of the FZD antibody was tested using biolayer interferometry as previously described (Tao et al., 2019).

### Electrophysiology

Whole-cell patch-clamp electrophysiology was performed on DA neurons at day 45 of differentiation. Cells grown on coverslips were submerged in a HEPES extracellular solution (140 mM NaCl, 1.3 mM CaCl_2_, 1 mM MgCl_2_, 2 mM KCl, 25 mM HEPES, 28 mM glucose and 325 mOsm) at room temperature. Intracellular recordings were made with a Multiclamp 700A amplifier (Molecular Devices) using borosilicate glass pipettes (4-8 MOhm) filled with a potassium-gluconate based intracellular solution (140 mM K-gluconate, 4 mM NaCl, 10 mM HEPES, 1 mM EGTA, 0.5 mM MgCl_2_•6H_2_0, 4 mM Mg-ATP, 0.5 mM Na_2_-GTP and 290 mOsm, pH adjusted to 7.2 with KOH). Data were acquired in current-clamp mode using pClamp 10.7 (Molecular Devices), with current low-pass filtered at 2 kHz and sampled at 10 KHz. Cells with neuronal morphology were selected based on round or oblong cell bodies with visible processes. Resting membrane potentials (ranging from −15 mV to −35 mV) were measured immediately after gaining whole-cell access to the cell. To measure excitability, cells were maintained at membrane potentials ranging from −60 mV to −90 mV, and current pulses ranging from +5 to +65 pA were injected for 500 ms at 5 pA increments to induce action potentials. To measure rebound action potentials, cells were held at a sub-threshold membrane potential (approximately −35 mV to −40 mV) and injected with a train of 500 ms −20 pA step currents.

### Animals

Female RNU nude rats were purchased from Charles River and permitted 2 weeks to acclimatize before surgery. Rats were housed individually with a 12 h/12 h light dark cycle and provided food and water *ad libitum*. On the day of surgery, the weight of the rats was between 193 and 234 g. Rats were carefully monitored daily for health by technicians in Terrence Donnelly Centre for Cellular & Biomolecular Research (Toronto, Canada). All procedures were conducted in accordance with University Animal Care Committee and were approved by the Office of Research Ethics at University of Toronto.

### 6-OHDA infusion and cell engraftment surgery

Rats were operated on under sterile conditions in a biosafety cabinet to ensure that the rats health was not compromised. The rats received the 6-OHDA lesion in the median forebrain bundle as described previously (Kirik et al., 1998). In brief, rats were induced for anesthesia using 5% isoflurane and maintained with ∼2% isoflurane while secured to stereotaxic frame (Kopf Instruments). The stereotaxic coordinates were calculated and adjusted for each animal for 6-OHDA injection (relative to bregma, AP=3.9, ML=1.2). At the coordinates, 8 mm holes were drilled in the skull overlying the right medial forebrain bundle. A 10 μl Hamilton syringe (Hamilton Company) was loaded in the pump with a 2 inch, 33 G needle, with a 45° tip angle (Hamilton Company). Hamilton syringe pumps were loaded with newly thawed (within 1 min) and tinfoil-covered 6-OHDA (Sigma Aldrich) stock solution, which was prepared at 8 µg/µl, in 0.9% sterile saline solution containing 0.02-0.04% of the antioxidant L-ascorbic acid. Hamilton syringe needles were directed to the median forebrain bundle (from dura, DV=7.7) and 6OHDA was microinjected (20 µg, 2.5 µl volume, 0.5 µl/min). Five minutes after the completion of microinjection, the needle was raised by 0.25 mm. One minute later, the needle slowly and completely retracted from the brain. For intrastriatal transplantation, F5L6.13 patterned VM NPCs were dissociated at day 16 and resuspended at 125,000 cells/µl in HBSS buffer solution (Sigma-Aldrich) containing 0.05% Dnase I (Sigma-Aldrich). Rats were prepared for surgery similar to 6-OHDA injection and a craniotomy was made overlying the striatum (AP=1.0, ML=2.5) and the dura was reflected. A sterile microinjector needle (26G, 7804-03, Hamilton Company) loaded with each cell suspension or buffer solution for only the sham-treated group. The microinjector needle was lowered into the striatum to a depth of 4.7 mm from dura through the craniotomy (∼1 mm/min). A total of 2.0 µl volume of the cell suspension was injected at 0.5 µl/min. The injection needle was left in place for 3 min after injection was complete and then slowly retracted from the brain over 2 min.

### Behavioral testing

Animals were tested for behavioral signs of impairment in amphetamine rotation tests (Kirik et al., 1998). All tests were conducted under observation with a video camera (Cannon, VIXIA HFR500) and data were saved for post processing. The rats were injected intraperitoneally with 5 mg/kg d-amphetamine (Tocris) and the rotation test was conducted in a plastic bucket with outward tapered walls (lower diameter=22 cm, upper diameter=33 cm, height=22 cm). Scoring for rotation was performed manually. Animals were scored only for full 360° turns left or right relative to a northern reference point. Net rotation was calculated as (right rotation−left rotation) over the course of a 20 min time frame.

### RNA-seq analysis

For bulk RNA sequencing, VM progenitors were patterned with either F5L6.13 or CHIR99021 to day 16. H1 hESC cells and VM progenitors were then lysed in Trizol (ThermoFisher Scientific), and RNA was extracted following the manufacturer's instructions. Sequencing libraries were prepared and run on an Illumina NextSeq-500 instrument at the Lunenfeld-Tanenbaum Research Institute Sequencing Facility (Toronto, ON), generating 75 bp single-read FASTQ files. The transcript reads from FASTQ files were aligned to the Ensembl GRCh38v94 human transcriptome and quantified using Kallisto (Bray et al., 2016), following default parameters. Principal component analysis and differential expression analysis were performed using the DESeq2 package with R software v3.6 (https://www.r-project.org/) using default parameters (Love et al., 2014).

### Single cell analysis

In preparation for scRNA sequencing, VM patterning was induced from H1 hESC using CHIR99021 or F5L6.13, and continued differentiation to day 11 and day 30. At the time of harvest, live cells were harvested using Accutase and washed three times before filtering for single cells with 40 µm cell strainers (Falcon). The cell preparations were then given to the Princess Margaret Genomic Sequencing Center (Toronto, Canada) for processing*.* In the scRNAseq experiments, a single biological replicate from the same differentiation and timepoint within the same batch was used in parallel, employing the 10x Cellplex platform. The CMO labeling and libraries were prepared according to the 10X protocol. Sequencing was carried out on an Illumina NovaSeq 6000, and data were aligned and processed using 10X Genomics Cell Ranger pipeline (version 6.0.0) to the Human reference (GRCh38)-2020-A.

Subsequent analysis was performed using Seurat v4.0 (https://satijalab.org/seurat/) in R software v4.1. Seurat objects were created with the cell-gene expression files generated from the CellRanger pipeline for each treatment. The Seurat objects generated from each timepoint (i.e. day 11 or day 30) were merged. Cells were filtered based on a 200 cut-off for minimum number of genes, 7500 for maximum of genes and less than 5% of mitochondrial expression. The gene expression of each cell was normalized using the global-scaling normalization method to normalize RNA expression measurements for each cell by the total expression, which was then multiplied by a scale factor of 10,000. Highly variable genes by cell were computed and the top 2000 features from each dataset were used for subsequent downstream clustering. An elbow plot was used to estimate the number of principle components required for UMAP embedding. This distance matrix was then reduced to low dimensionality using UMAP with the first 15 principal components dimensions and clusters identified using a resolution of 0.5. For differential gene expression between clusters, the FindMarkers function was used with a comparison of the indicated clusters. GSEA was performed with GSEA software (Broad Institute) using a ranked gene expression list based on Log2Fold change for day 11 cluster 0 versus clusters 1, 2 and 5. The GO:0045746 (Notch), GO:0000165 (MAPK), M5923 (PI3K_AKT) gene sets were used for GSEA extracted from MSigDB. For all subsequent analyses, meta, filtered and normalized data were exported from Seurat for integration in various R packages. The CellCycleScoring was performed using the list of signature genes from (Tirosh et al., 2017). UCell gene signature scoring was performed by following https://github.com/carmonalab/UCell. The list of gene markers used for UCell signature scoring is listed in Table S3. The scRNA datasets were prepared for the ShinyApp using the ShinyCell package (Ouyang et al., 2021) and deployed on shinyapp.io, which can be accessed at https://andyydh.shinyapps.io/scRNA_VM_progenitor/.

### HiDDEN analysis

HiDDEN clustering analysis was performed on the 3990 DA^+^ neurons from the combined CHIR and F5L6 populations (Fig. 5E). The original labels assigned to CHIR and F5L6 cells were 0 and 1, respectively. The HiDDEN model training was carried out in python following the published github code from https://github.com/tudaga/LabelCorrection. Dimensionality reduction was performed using principal component analysis. HiDDEN Continuous Scores were derived as the fitted conditional probability of label 1 given the gene expression profile for each cell computed by training a logistic regression. The optimal number of principal components (*P*=55) to use in the logistic regression was determined using the Kolmogorov–Smirnov heuristic (Goeva et al., 2023 preprint). The resulting continuous scores were binarized using k-means clustering (k=2) to define new binary per-cell labels. All 2407 CHIR cells retained label 0 (new label CHIR_L0). The 1583 F5L6 cells were split into two groups based on the HiDDEN. The subset with expression profiles similar to CHIR cells was labeled F5L6_L0 and the subset with a transcriptional difference from CHIR cells was labeled F5L6_L1. Differential expression analysis on the populations identified by HiDDEN was performed in scanpy using Wilcoxon rank-sum test. Significantly enriched or depleted genes were defined based on a threshold of adjusted *P*<0.05 and the sign of the log fold-change in expression (Fig. S6I). GO term enrichment analysis of the 394 genes enriched in F5L6_L1 versus L0 (combined CHIR_L0 and F5L6_L0) was performed using the web tool PANTHER Overrepresentation Test (Released in 20221013) to identify significant GO biological process terms using Fisher's Exact test and a Bonferroni multiple testing correction with adjusted *P*< 0.05. Dot plots in Fig. S6J were produced in scanpy based on the HiDDEN-refined binary labels and the log-normalized scaled gene expression.

### Statistics

All statistical methods are explained in the figure legends. Data were analyzed using GraphPad Prism (version 8) software in at least three independent experiments. Unless stated otherwise, values are shown as mean±s.e.m and asterisks in figures indicate significance according to a two-tailed Student's *t*-test between two groups (*P≤0.05, **P≤0.01).

## Supplementary Material



10.1242/develop.202545_sup1 Supplementary information

## The people behind the papers

This article has an associated ‘The people behind the papers’ interview with some of the authors.
