# Supplementary material for: Exploiting spatiotemporal regulation of FZD5 during neural patterning for efficient ventral midbrain specification
Source: Development. 2024 Mar 4;151(5):dev202545. doi: 10.1242/dev.202545 (PMC10946437; doi:10.1242/dev.202545)
Supplement: Supplementary information [file develop-151-202545-s1.pdf]

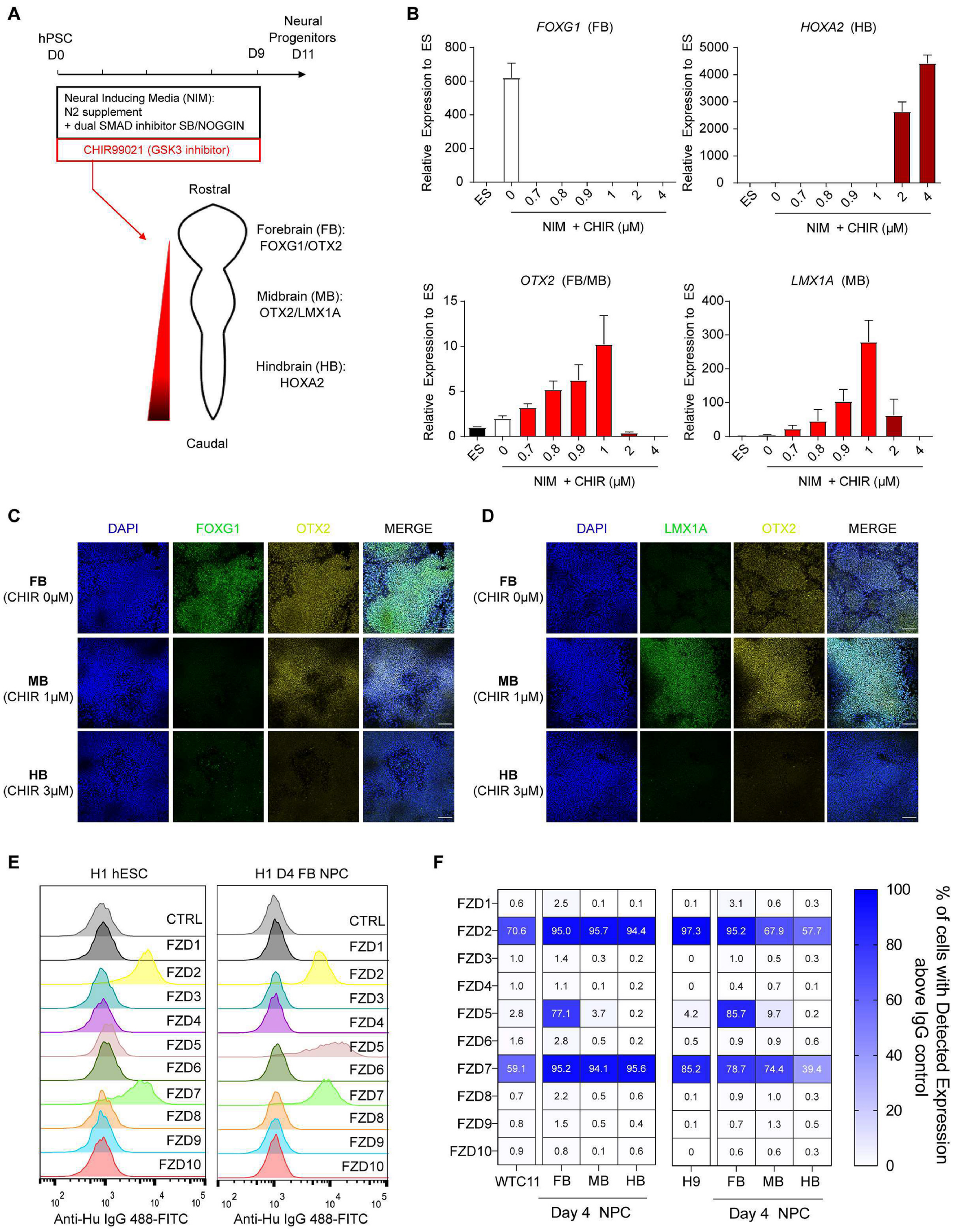

Fig. S1. Related to Fig. 1.

A. Overview of differentiation protocol of regional neural progenitors. **B.** Gene expression of regional specific markers was measured by qPCR at day 11 of differentiation treated with various concentration of CHIR99021 patterned from Hl hESC. The gene expression was normalized to the non-differentiated ES, Mean ± s.d. of 3 technical replicates from 1 experiment. Representative of 3 independent experiments. **C-D.** Immunofluorescence staining of FOXG 1/0TX2 (C) and LMX1A/OTX2 **(D)** in patterned neural progenitors at day 11. Representative of 3 independent experiments . Scale bar, 100 μm. E. Histogram of flow cytometry staining of FZD receptors in Hl ES cells and Day 4 FB NPC. **F.** The percentages of each cell surface FZD expression in WTCl 1 (hiPSC) and differentiated day 4 regional NPCs, and H9 ESC and differentiated day 4 regional NPCs. Representation of 3 independent experiments.

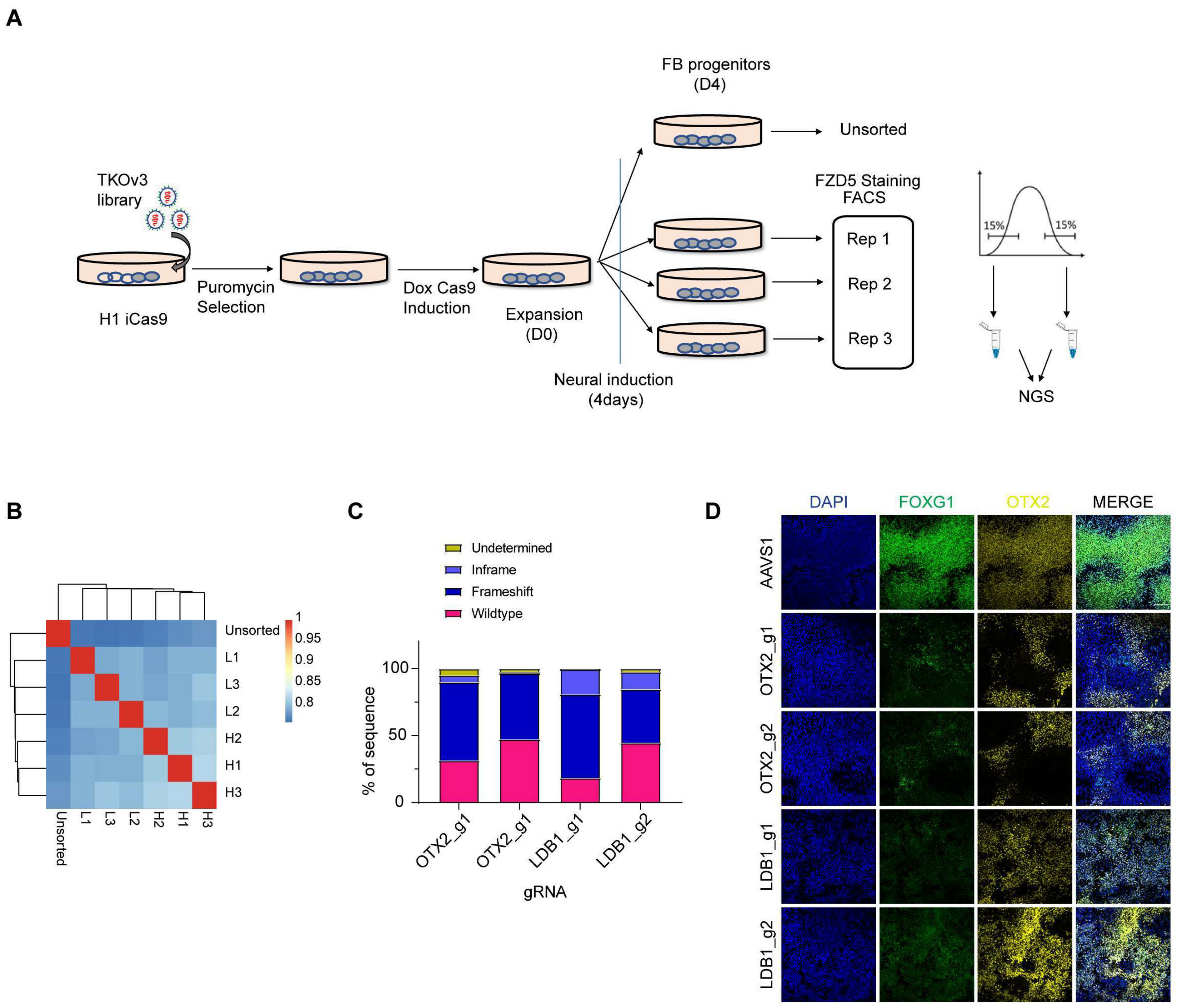

Fig. S2. Related to Fig. 2.

A. Schematic outlining the CRISPR screen in Hl -iCas9 cells. **B.** Pearson correlation analysis of the gRNA counts in each sample. L denotes the FZD5-low sorted population while H denotes FZD5-high sorted, and the numbers represent the replicates. C. KO efficiency of gRNAs targeting OTX2 and LDBl cells as determined by TIDE sequencing analysis. **D.** Immunofluorescence staining of FOXG 1 and OTX2 in day 11 FB patterned *AAVS J-KO* cells compared to *OTX2-KO* cells and *LDBl -KO* cells. Representative of 3 independent experiments. Scale bar, 100 µm.

Development • Supplementary information

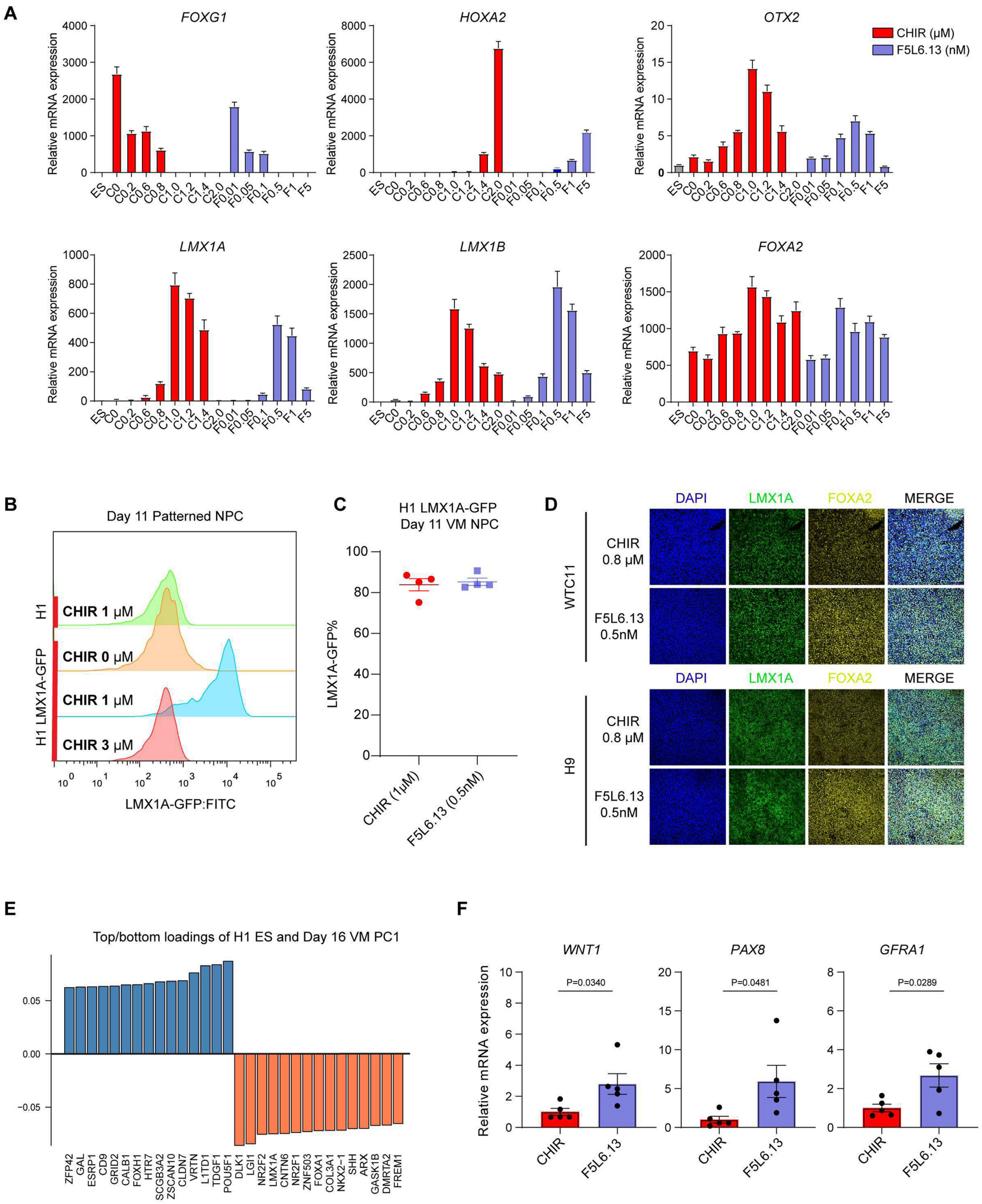

Fig. S3. Related to Fig. 3.

A. Dose response of CHIR99021 and F5L6.13 for neural patterning. The gene expression was normalized to the non-differentiated ES. Mean + s.d. of 3 technical replicates from 1 experiment. Representative of 3 independent experiments. **B.** Histogram of GFP expression in day 11 NPC differentiated in Hl hESC parental line with 1 uM CHIR9901 and Hl hESC LMX l A-GFP reporter line with 0 µM, 1 µM or 3 µM CHIR9901. Representative of 3 independent experiments. C. Percentage of LMX l A-GFP expressing cells differentiated from a Hl hESC LMX l A-GFP Reporter line with 1 µM CHIR99021 or 0.5 nM F5L6.13. Data are represented from 4 independent experiments. Mean ± s.e.m. **D.** Immunostaining of VM markers LMX1A/FOXA2 at day 16 patterned from WTCl 1 hiPSC and H9 hESC. Images are representative of 3 independent experiments. Scale bar, 100 µm. E. Top 15 gene loadings of PC l which explains the variance between the Hl ESC and day 16 VM patterned NPC groups. **F.** qPCR gene expression of *PAX8/ WNTJIGFRAJ* at day 16 differentiated cells patterned with CHIR99021 or F5L6.13. Mean ± s.e.m. n = 5 independent experiments, two-sided t-test. P-value are indicated on graph.

Development • Supplementary information

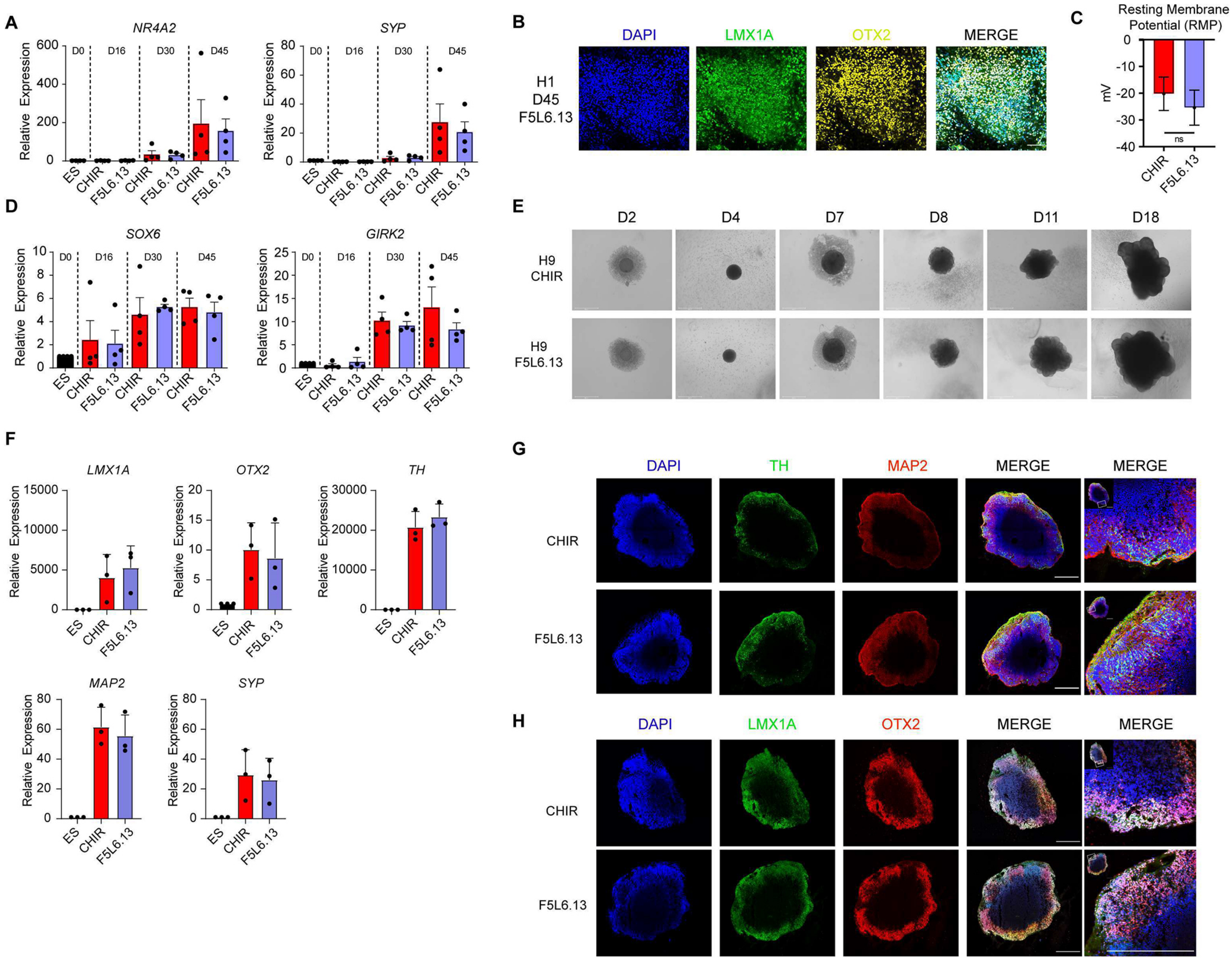

Fig. S4. Related to Fig. 4.

A. Time course qPCR gene expression of *NR4A2* and *SYP* in CHIR99021 or F5L6.13 patterned cells. Mean ± s.e.m. n = 4 independent experiments. **B.** Immunostaining of LMX IA/FOXA2 on D45 differentiated neuronal cultures from VM progenitors patterned with F5L6.13. Images are representative of 3 independent experiments. Scale bar, 100 µm C. Resting membrane potential of patched neurons from CHIR99021 and F5L6.13 patterned neuronal cultures. Mean ± s.e.m. n = 9 for CHIR99021 , n = 13 for F5L6.13 **D.** Time course qPCR gene expression of *SOX6* and *GIRK2* in CHIR99021 or F5L6.13 patterned cells. Mean ± s.e.m. n = 4 independent experiments. E. Phase contrast image of the midbrain organoids patterned from H9 hESC at the timepoints indicated. Images are representative of 3 independent experiments. Scale bar, 650 µm **F.** qPCR gene expression of *LMXJ A, OTX2, TH, SYP,* and *MAP2* in D30 midbrain organoids patterned with 0.8 µM CHIR9901 or 0.5 nM F5L6 .13 in H9 hESC. Mean ± s.e.m. n = 3 independent experiments. G. Immunostaining of TH/MAP2 (N) LMX IA/OTX2 of D30 sectioned midbrain organoids patterned with 0.8 µM CHIR9901 or 0.5 nM F5L6.13. Images are representative of 3 independent experiments. Scale bar, 500 µm.

Development • Supplementary information

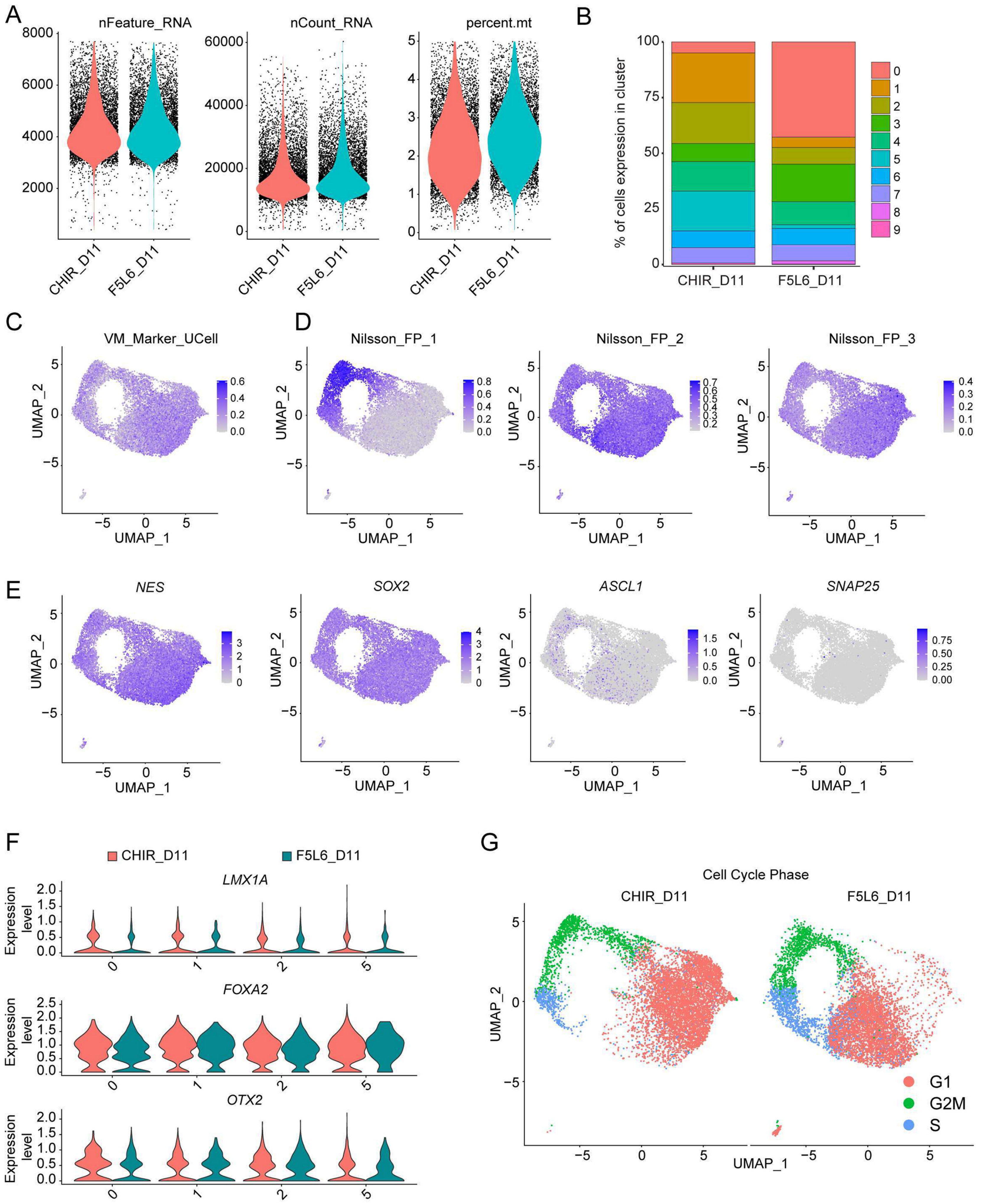

Fig. S5. Related to Fig. 5.

A. Violin plots displaying distribution of expression levels of feature counts, transcript counts and percentage of mitochondria genome across all cells after filtering. **B.** Proportion of clusters identified in each treatment condition. C. UCell scoring for simultaneous assessment of the expression level of several genes known for VM marker across all cells (See Table S3 for the list). Colored according to scaled expression by cell. **D.** UCell scoring for expression of genes representing distinct floor plate marker clusters identified from (Nilsson et al., 2021). Colored according to scaled expression by cell. E. Normalized expression levels of *NES, SOX2, ASCL J , SNAP25* superimposed onto the UMAPs. Colored according to expression levels by cell. **F.** Violin plots displaying distribution of expression levels of *LMX J AIFOXA2/ 0TX2* across clusters 0, 1, 2, 5 separated by treatment condition. G. UMAP plots showing the cell cycle status of the clusters separated by treatment conditions. G2M and S cell cycle status were classified using the function CellCycleScoring in the Seurat package.

Development • Supplementary information

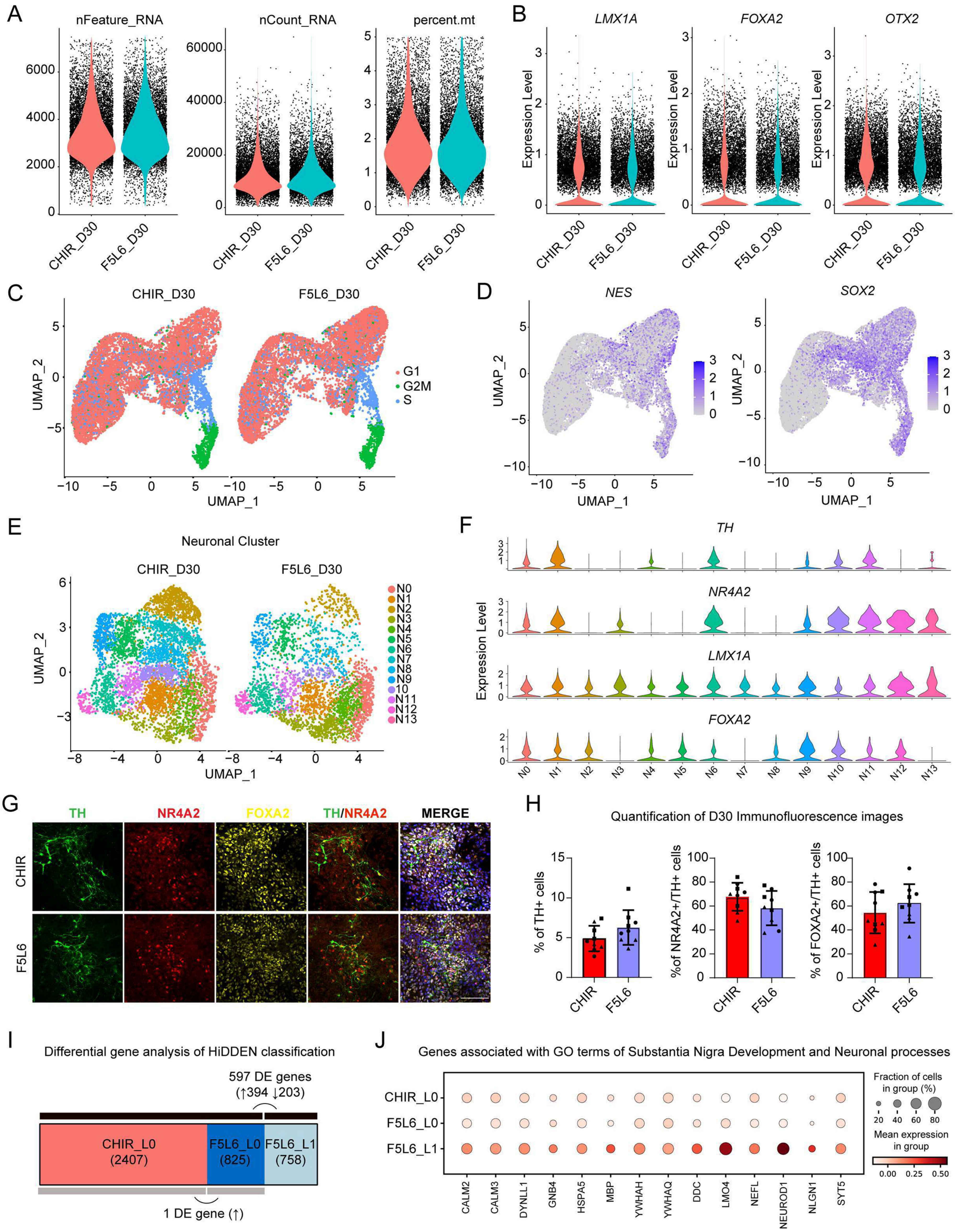
A
nFeature_RNA
nCount_RNA
percent.mt
CHIR_D30
F5L6_D30
B
LMX1A
FOXA2
OTX2
Expression Level
C
CHIR_D30
F5L6_D30
UMAP_1
UMAP_2
G1
G2M
S
D
NES
SOX2
E
Neuronal Cluster
N0
N1
N2
N3
N4
N5
N6
N7
N8
N9
10
N11
N12
N13
F
TH
NR4A2
LMX1A
FOXA2
G
TH
NR4A2
FOXA2
TH/NR4A2
MERGE
CHIR
F5L6
H
Quantification of D30 Immunofluorescence images
% of TH+ cells
%of NR4A2+/TH+ cells
% of FOXA2+/TH+ cells
I
Differential gene analysis of HiDDEN classification
597 DE genes
(↑394 ↓203)
CHIR_L0 (2407)
F5L6_L0 (825)
F5L6_L1 (758)
1 DE gene (↑)
J
Genes associated with GO terms of Substantia Nigra Development and Neuronal processes
CHIR_L0
F5L6_L0
F5L6_L1
Fraction of cells in group (%)
Mean expression in group
CALM2
CALM3
DYNLL1
GNB4
HSPA5
MBP
YWHAH
YWHAQ
DDC
LMO4
NEFL
NEUROD1
NLGN1
SYT5

Fig. S6. Related to Fig. 6.

A. Violin plots displaying distribution of expression levels of feature counts,

transcript counts and percentage of mitochondria genome across all cells after filtering. **B.** Violin plots displaying distribution of expression levels of *LMX J AIFOXA2/ 0TX2* across all cells. C. UMAP plots showing the cell cycle status of the clusters separated by treatment conditions. **D.** Normalized expression levels of *NES* and *SOX2* superimposed onto the UMAPs. Colored according to expression levels by cell. E. UMAP plot showing the cell identity of treatment conditions contributing the subclustered neuronal population. **F.** Violin plots displaying distribution of expression levels of *TH, NR4A2 , LMX J A ,* and *FOXA2* in neuronal clusters. G. Immunostaining of TH/NR4A2/FOXA2 on day 30 differentiated neuronal cultures from VM progenitors patterned with CHIR99021 or F5L6.13. Scale bar, 100 µm. **H.** Quantification of TH+ cells over DAPI nuclei staining, TH+ cells expressing NR4A2 and TH+ cells expressing FOXA2. Three images were taken at random locations per condition (represented by each symbol) and three independent experiments were used for counting. **I.** Schematic outlining the differential gene expression analysis from the HiDDEN clusters. **J.** Dot plot highlighting the genes associated to the gene ontology terms associated with substania nigra development and neuronal processes across the HiDDEN clusters.

Development • Supplementary information

Table S1. Regulators of FZD5 cell surface expression in differentiated anterior neural progenitor cells identified in CRISPR screen.

Positive Regulators				
FZD5	PAN2	AMER1	KIF7	EMC4
FUT8	SAP30	TEAD1	LCMT1	GET4
SLC35C1	FZD7	BAP1	PDS5B	VMA21
CTNNBIP1	USP46	DET1	FLII	MSN
AXIN1	STK11	PSIP1	BRD2	RERE
DOT1L	SUZ12	MED24	LBH	CADPS2
LDB1	SIX3	STRAP	PHIP	RIC8A
PTK7	AGO2	SPTLC1	OTX2	NOB1
MED12	MED23	MLLT1	TM2D3	SRP14
PPP6C	BRPF1	TCF7L1	EMC2	MBTPS2
MED25	TSC2	TSC1	TP53	PLCG1
MED13L	PCGF3	JMJD6	RALBP1	GLTSCR2
MED16	EED	SEC63	PHF6	SMARCC1
KAT6A	JTB	IFT122	TMOD3	CHP1
TGFBR2	EMC1	ZNF507	AP1G1	RHOA
NHLRC2	ZFAND2B	DDX3X	MED15	TEAD2
PAXIP1	SLC35B2	MEN1	SMARCD1	
MLLT10	CIC	C1GALT1C1	TOE1	
MMGT1	FXN	RUNX1T1	BAHD1	
Negative Regulators				
PKDCC	GREB1L	RFX7	PPIH	RALGAPB
NF2	CBX5	LATS2	C19orf43	DDX6
DVL2	PAX6	STAM	ARIH2	NEDD4L
CARM1	DCP2	SLMAP	CCND2	MYCN
RAX	HECTD1	HTT	SCAF8	
RCOR2	RPS6KA3	STUB1	EDC4	
SOX11	ZEB2	RBM26	C20orf173	
HGS	GPC4	MBD4	UBE3B	
AP2S1	SMARCA5	E2F4	IREB2	
SPOP	TLE4	FLCN	UBTD2	
UBE2H	SMG9	NFKBIZ	EDC3	
JARID2	CDK13	ZNF496	SOX4	
FUS	ZMYND8	BAZ1B	CTNNB1	
RARA	TAOK1	KRAS	CYP51A1	

Table S2. Marker genes used for UCell scoring.

VM_Marker	Nilsson_FP_1	Nilsson_FP_2	Nilsson_FP_3
LMX1A	HMGB2	DLK1	ABCA8
LMX1B	UBE2C	MDK	CMTM8
FOXA2	CENPF	TPBG	SULF1
OTX2	HISTIH4C	SDC2	TFF3
SHH	PTTG1	VIM	IGFBP2
CORN	PCLAF	COL1A2	SPON1
	TOP2A	CCND1	RFX4
	CKS1B	FABP7	XBP1
	NUSAP1	RCN1	CMTM7
	SMC4	PITX2	IGDCC3

Table S3. Differential expressed genes identified comparing label 0 to label 1 of DA neurons in HiDDEN analysis, related in Figure 6.

Label 0 upregulated:

DMRTA2	LMO3	ZFHX3	TLE4	DLK1	FTH1	RPS8
APP	RPL11	PTPRO	RPS13	PLXDC2	RND3	RPL10
RPL37A	RPL13A	RPS11	RPS3A	RPS14	RPL9	RPL22
RPL18A	RPS15A	RPL21	RPL7	RPS27	RPL27A	IGFBP2
RPL3	RPL6	RPL32	RPL18	EEF1G	RPL15	RPL28
ADCYAP1	NOVA1	RPS19	RPL3	RPL6	RPL32	RPL18
NACA	RPL37	HMGN1	TH	MIR99AHG	RPL36	RPS16
FAT3	LINC00461	PPP1R14B	SOX2-OT	TRIM9	TOMM7	ZFHX4
RPS18	HNRNPC	DCC	SPOCK1	RPL7A	RPL35A	PCSK2
RPS6	SLITRK6	ATP5F1E	RPLP0	RACK1	NR2F2	ALCAM
NCALD	RPS9	RPL30	SLC39A10	ZNF503	RPL26	RPL4
PKIB	RPL31	TUBA1B	SOX11	NEDD4L	PIK3R1	EIF3L
NREP	VKORC1	RPL29	ANKRD12	NTM	HES6	RPS5
TPBG	YBX1	NUCKS1	RPL36A	AL049637.2	TAGLN2	GULP1
MTPN	MINDY2	CHL1	RPS15	RPL19	CHN2	DAAM1
AP000894.2	EIF3E	COX7C	QDPR	FUT8	LBH	ROBO1
STARD4-AS1	RPS21	DDX46	FXR1	CUX1	DCBLD2	RPL10A
PDZRN4	SEZ6L	SNHG29	RMST	NECAB1	RPL14	SKA2
CTCF	SLC2A13	FTL	PABPC1	C21orf62	PFN2	OTX2
EPHA4	RPL41	NTN1	EIF3F	RTF1	RPS7	HMGN2
PTTG1IP	FAM110B	ZNF292	SSH2	PITX2	HS3ST5	POSTN
PLS3	NPAS3	RPL13	EEF1A1	RPL5	TUBB	NR4A2
CHD4	CAMK2N1	RPS12	RPL34	RPS27A	SOX2	RPL17
DDX5	TMA7	RPS4X	RPS23	TPT1	PCDH17	WASF3
CKB	RPL8	ADCYAP1	NOVA1	RPS19	CIRBP	IGFBP5
TUBB3	RPS10	NACA	RPL37	HMGN1	CADM1	CHD7
SLIT2	FNBP1L	EEF1G	RPL15	RPL28	CELF4	PAWR
GNAI2	GDAP1L1	RPL38	RPS24	RPLP2	PON2	STMN1
SVBP	POU3F2	LRRC4C	REEP1	RPL39	RPSA	PFDN5
RPS17	RPL23A	RPL23	ACTG1	CXXC4	OBI1	DIPK2A
RPS29	RPS28					

Label 1 upregulated:

CRNDE	IRX3	LMO4	NEUROD1	RALYL	SSBP2	EPHA5
PTPRN	BTBD3	NR2F1	TPH1	SCG2	FOXP2	AC010247.2
SYT13	MAP1LC3A	LIMCH1	C1GALT1	PCSK1	RPLP1	MAB21L1
DACH1	VGF	DUSP4	SEPTIN6	BEX3	EPHA5-AS1	RXRG
DDC	TENM1	PTPRK	TMSB10	GRIN2B	RUNX1T1	NLGN1
PAM	CASC15	MBP	CYSTM1	BEX5	CYCS	BEX1
LNPK	AFF3	NEFM	MAD1L1	NRXN1	ATP6V0C	VAT1
CPE	SARAF	LUZP2	STXBP1	WNT4	PRKACB	SEC11C
ELAVL2	DNAJA1	ANKRD29	ONECUT3	TCEAL5	RGMA	FIGN
TMSB4X	RTN4	ELL2	FOS	HSP90B1	VIM	ID2
NEFL	GLRX	ATP1A3	LRRC3B	HSPE1	COTL1	DOK5
PKIA	HTR2A	GNAI1	IGFBPL1	BCL11A	ARMCX3	SYAP1
BMERB1	BEX2	SHC4	TSC22D3	TRAPPC5	TXN	AKAP7
PDE10A	TLE2	AKAP12	SOX4	GSTM3	CENPV	GNAL
YWHAH	TRA2B	QKI	BAALC	PLD3	AAK1	UNCX
NDUFB8	UBE2QL1	NME1	COX8A	ARID5B	TSPYL4	ARMH4
GNG4	DYNLL1	FNDC5	GNB4	EIF5	TTC39C	CLTC
CNST	PPA1	SRGAP3	GABBR2	MAP4K5	SIK3	PDHA1
CRYBG3	CDKN2D	UBL3	STMN2	COX7B	MBNL2	CTNNA2
NCS1	PITPNC1	SGIP1	MORF4L2	GNG3	DSTN	RPS26
ANK2	ATP6V0E2	GRM7	SPAG9	KCNIP4	CRIM1	SEMA3A
HSPA8	DGKD	SYT5	HMGA1	PARD6A	RANGAP1	SCOC
GNB2	KCNJ6	ATP1B3	LYPD1	CAST	MGAT4C	MICOS13
TLK1	SEMA6A	PJA2	ARPC5	CHML	SNRPB	MAL2
ATP5MC1	NSG2	CCDC184	CNTNAP5	PTPRD	SAT2	YWHAQ
PTMA	NRIP3	SEPTIN11	USP48	HSPD1	BLOC1S1	SNHG8
MAP2	RTN1	DDX21	SELENOK	KLHL35	MLLT3	KCNMA1
TES	RAB10	ARG2	EML6	ATP1A1	CYC1	REEP5
RAB9B	SLC22A17	ANK3	LONRF2	HSPH1	COX7A2	RND2
FGF12	CALY	SH3KBP1	GLIS3	S100A	PHLDA1	IRX5
SNAP25	ID4	EPS15	CSRP2	TUNAR	SPCS2	ARX
CNR1	OSBPL1A	KIF21A	DCLK1	JAK1	PLXNA4	SLC7A8
CARTPT	TBCA	DMAC1	POU2F2	ASPHD1	TCEAL7	NUCB2
CNTNAP3B	NPTX1	TMEM132B	AFF2	SFMBT2	CALB1	HPGD
PROX1	ITPRID2	PPFIA2	EIF1	UCHL1	KPNA2	PRKAR1A
FTO	MARK1	ADGRL2	CALR	NDUFA13	ITGB1	CDH8
POU6F2	CASK	PALM2-AKAP2	PCDH10	ATP6V1G1	RGS7	PCB
CHD5	MTURN	IGSF21	GALNTL6	NDUFA5	NAV3	

Development • Supplementary information

Table S4. List of qPCR primers used in this study.

Oligo Name	Sequence (5' to 3')
FOXA2.qPCR.Fwd	CCGTTCTCCATCAACAACCT
FOXA2.qPCR.Rev	GGGGTAGTGCATCACCTGTT
FOXG1.qPCR.Fwd	AGAAGAACGGCAAGTACGAGA
FOXG1.qPCR.Rev	TGTTGAGGGACAGATTGTGGC
FZD5.qPCR.Fwd	ACACCCGCTCTACAACAAGG
FZD5.qPCR.Rev	CTCAGGATAGCGGAAGCGTT
GFRA1.qPCR.Fwd	GAACCCCTAAAAGCGGAACC
GFRA1.qPCR.Rev	TCAGCTCCATCCAGTGAAAG
HOXA2.qPCR.Fwd	CGTCGCTCGCTGAGTGCCTG
HOXA2.qPCR.Rev	TGTCGAGTGTGAAAGCGTCGAGG
LMX1A.qPCR.Fwd	CGCATCGTTTCTTCTCCTCT
LMX1A.qPCR.Rev	CAGACAGACTTGGGGCTCAC
LMX1B.qPCR.Fwd	CTTAACCAGCCTCAGCGACT
LMX1B.qPCR.Rev	TCAGGAGGCGAAGTAGGAAC
MAP2.qPCR.Fwd	CCGTGTGGACCATGGGGCTG
MAP2.qPCR.Rev	GTCGTCGGGGTGATGCCACG
OTX2.qPCR.Fwd	ACAAGTGGCCAATTCACTCC
OTX2.qPCR.Rev	GAGGTGGACAAGGGATCTGA
PAX8.qPCR.Fwd	ATAGCTGCCGACTAAGCATTGA
PAX8.qPCR.Rev	ATCCGTGCGAAGGTGCTTT
PPIB.qPCR.Fwd	GGAGATGGCACAGGAGGAA
PPIB.qPCR.Rev	GCCCGTAGTGCTTCAGTTT
SYP.qPCR.Fwd	ACCTCGGGACTCAACACCTCGG
SYP.qPCR.Rev	GAACCACAGGTTGCCGACCCAG
TH.qPCR.Fwd	CGGGCTTCTCGGACCAGGTGTA
TH.qPCR.Rev	CTCCTCGGCGGTGTACTCCACA
WNT1.qPCR.Fwd	GAGCCACGAGTTTGGATGTT
WNT1.qPCR.Rev	TGCAGGGAGAAAGGAGAGAA
NR4A2.qPCR.Fwd	CAGGCGTTTTCGAGGAAAT
NR4A2.qPCR.Rev	GAGACGCGGAGAACTCCTAA
SYP.qPCR.Fwd	ACCTCGGGACTCAACACCTCGG
SYP.qPCR.Rev	GAACCACAGGTTGCCGACCCAG
SOX6.qPCR.Fwd	AGGGAGTCTTGCCGATGTG
SOX6.qPCR.Rev	CAGGCTCTCAGGTGTACCTTTA
GIRK2.qPCR.Fwd	CACATCAGCCGAGATCGGAC
GIRK2.qPCR.Rev	GGTAGCGATAGGTCTCCCTCA
MAP2.qPCR.Fwd	CCGTGTGGACCATGGGGCTG
MAP2.qPCR.Rev	GTCGTCGGGGTGATGCCACG

Development • Supplementary information

Table S5. List ofPCR primers and gRNA sequences used in this study.

CRISPR-Cas9 Knockout

gRNA Targeting	gRNA seqenuence
AAVS1	GGGGCCACTAGGGACAGGAT
OTX2_g1	GGAGAGGACGACGTTCACTC
OTX2_g2	AAATCAACTTGCCCGAGTCG
LDB1_g1	GTGGTCGGAGACTCACTCGG
LDB1_g2	GAGATGCTGCGGAAGTAGCGT

TIDE PCR primers

	TIDE PCR FWD Primer	TIDE PCR REV Primer
AAVS1	CCTGTCATGGCATCTTCCAG	CAAGTCTCCCTCCCAGGAT
OTX2_g1	GATGGAGGAACAAAGGCCCA	ATTAAGTGGTGACGGGCAGG
OTX2_g2	GATGGAGGAACAAAGGCCCA	ATTAAGTGGTGACGGGCAGG
LDB1_g1	TCCTTGCCATGCATGTGAGT	CCCAGGTCCCCTATTCTCCA
LDB1_g2	AGGCACACACCATATGGCAA	CACTGGGAGAGAGGATGGGA

TIDE Sequencing Primers

	TIDE Sequencing Primer
AAVS1	CTATGTCCACTTCAGGACAGCATG
OTX2_g1	GGCAGAAGGAGAATAGTTTCC
OTX2_g2	GGCAGAAGGAGAATAGTTTCC
LDB1_g1	GTGGGGAAAACTTCAAAAGG
LDB1_g2	GCCCTTCTTATTTTGCCAGT

Homology Arm PCR Primers

	Sequences
LMX1A LHA - FWD	TGAAGGTAGGCACCTAACC
LMX1A LHA - REV	CTCCTGGACGTAGCCTTC
LMX1A RHA - FWD	TCCATACCGACGATCTGCG
LMX1A RHA - REV	GCAGATGCTGAAGCTCAAG

Nickase Sequences

	Sequences
Nickase 1	CTAGAGTTCTGTGACTAGGC
Nickase 2	CATATTCTTTGAGGGGTCAC

Development • Supplementary information

Table S6. List of antibodies used in this study.

**Primary Antibody**	**Antibody Cat No.**	**Species**	**Dilution**
FOXG1	Abcam (ab18259)	Rabbit	1:400
LMX1A	Millipore AB10533	Rabbit	1:1000
TH	Pel-Freeze P40101-150	Rabbit	1:500
TH	Millipore AB152	Rabbit	1:1000
FOXA2	R&D Systems AF2400	Goat	1:1000
OTX2	R&D Systems AF1979	Goat	1:1000
EN1	DSHB 4GII	Mouse	1:20
MAP2	ThermoFisher (13-1500)	Mouse	1:500
TUBB3	ThermoFisher (MA1-118)	Mouse	1:500
hNCAM	Santa Cruz sc-106	Mouse	1:500
NR4A2	ThermoFisher (MA1-195)	Mouse	1:1000
**Secondary Antibody**	**Antibody Cat No.**	**Species**	**Dilution**
Donkey anti-Rabbit IgG (H+L) Highly Cross-Adsorbed Secondary Antibody, Alexa Fluor 488	ThermoFisher (A-21206)	Donkey	1:250
Donkey anti-Mouse IgG (H+L) Highly Cross-Adsorbed Secondary Antibody, Alexa Fluor 594	ThermoFisher (A-21203)	Donkey	1:250
Donkey anti-Goat IgG (H+L) Cross-Adsorbed Secondary Antibody, Alexa Fluor 647	ThermoFisher (A-21447)	Donkey	1:500
Goat anti-Mouse IgG (H+L) Cross-Adsorbed Secondary Antibody, Alexa Fluor™ 488	ThermoFisher (A-11001)	Goat	1:500
Goat anti-Mouse IgG (H+L) Cross-Adsorbed Secondary Antibody, Alexa Fluor 568	ThermoFisher (A-11004)	Goat	1:500

Development • Supplementary information
