# Supplementary material for: Exploiting spatiotemporal regulation of FZD5 during neural patterning for efficient ventral midbrain specification
Source: Development. 2024 Mar 4;151(5):dev202545. doi: 10.1242/dev.202545 (PMC10946437; doi:10.1242/dev.202545)
Supplement: Supplementary file 1 [file develop-151-202545_review_history.pdf]

Exploiting spatiotemporal regulation of FZD5 during neural patterning for efficient ventral midbrain specification

Andy Yang, Rony Chidiac, Emma Russo, Hendrik Steenland, Quinn Pauli, Robert Bonin, Levi L Blazer, Jarrett J Adams, Sachdev S Sidhu, Aleksandrina Goeva, Ali Salahpour and Stephane Angers
DOI: 10.1242/dev.202545

Editor: James Briscoe

Review timeline

Original submission:	14 November 2023
Editorial decision:	21 December 2023
First revision received:	11 January 2024
Editorial decision:	2 February 2024
Second revision received:	5 February 2024
Accepted:	6 February 2024

Original submission

First decision letter

MS ID#: DEVELOP/2023/202545

MS TITLE: FZD5 cell surface expression is dynamically regulated during neural patterning

AUTHORS: Andy Yang, Rony Chidiac, Emma Russo, Hendrik Steenland, Quinn Pauli, Robert Bonin, Levi L Blazer, Jarrett J Adams, Sachdev S Sidhu, Alexandrina Goeva, Ali Salahpour, and Stephane Angers

I have now received all the referees' reports on the above manuscript, and have reached a decision. The referees' comments are appended below, or you can access them online: please go to BenchPress and click on the 'Manuscripts with Decisions' queue in the Author Area.

As you will see, the referees express considerable interest in your work, but have some criticisms and recommend a revisions before we can consider publication. The points the referees raise appear to be straightforward to address and mainly call for clarifications. I agree with Referee 2 that altering the title to reflect the ventral midbrain focus of the work would probably encourage more people to read your study.

If you are able to revise the manuscript along the lines suggested, which may involve further experiments, I will be happy to receive a revised version of the manuscript. Your revised paper will be re-reviewed by one or more of the original referees, and acceptance of your manuscript will depend on your addressing satisfactorily the reviewers' major concerns. Please also note that Development will normally permit only one round of major revision. If it would be helpful, you are welcome to contact us to discuss your revision in greater detail. Please send us a point-by-point response indicating your plans for addressing the referees' comments, and we will look over this and provide further guidance.

Please attend to all of the reviewers' comments and ensure that you clearly highlight all changes made in the revised manuscript. Please avoid using 'Tracked changes' in Word files as these are lost in PDF conversion. I should be grateful if you would also provide a point-by-point response detailing

© 2024. Published by The Company of Biologists under the terms of the Creative Commons Attribution License (https://creativecommons.org/licenses/by/4.0/).

how you have dealt with the points raised by the reviewers in the 'Response to Reviewers' box. If you do not agree with any of their criticisms or suggestions please explain clearly why this is so.

Reviewer 1

Advance summary and potential significance to field

Yang and colleagues have uncovered a novel mechanism of WNT signalling regulation via differential expression of WNT receptors, Frizzleds (FZDs), during pluripotent stem cell differentiation. They take advantage of this new knowledge to specifically active one (FZD5) of the 10 FZD receptors using a tetravalent antibody they have developed. The results of specifically activating FZD5 instead of generically activating WNT signalling GSK3beta inhibitors were tremendous. Specification of true substantia nigra neurons was significantly increased and were of greater maturity. This is a major leap forward in the understanding of ventral mibrain patterning, and provides a practical tool to improve differentiation of transplantable cells for Parkinson's.

Comments for the author

Yang and colleagues have uncovered a novel mechanism of WNT signalling regulation via differential expression of WNT receptors, Frizzleds (FZDs), during pluripotent stem cell differentiation. They take advantage of this new knowledge to specifically active one (FZD5) of the 10 FZD receptors using a tetravalent antibody they have developed. The results of specifically activating FZD5 instead of generically activating WNT signalling GSK3beta inhibitors were tremendous. Specification of true substantia nigra neurons was significantly increased and were of greater maturity. This is a major leap forward in the understanding of ventral mibrain patterning, and provides a practical tool to improve differentiation of transplantable cells for Parkinson's.

Some minor comments to address include:
1. It's not accurate to state “OTX2 and LDB1 are part of a gene regulatory network restricting FZD5 expression within anterior neural progenitors” since the deletion of OTX1 or LDB1 causes a failure of differentiation, and therefore a downstream effect could be loss of FZD5 expression I.e. it may not have anything to do with a ‘gene regulatory network’.

2. 269 up and 72 down genes when F5L6.13 is compared to CHIR99021 mDA cells for bulk RNAseq. Can KEGG pathway or GO analysis been performed on these gene lists?

3. What’s the evidence that “Cellular heterogeneity inevitably hampers engraftment efficiency or the generation of functional cells”? As long as some correctly patterned cells are in the mixture, the transplants promote recovery.

4. Set-up a ShinyApp of all scRNAseq data for others to easily analyse data.

5. Have CHIR99021-induced mDA cells been transplanted in the 6-OHDA model and how does functional recovery compare to F5L6.13-induced mDA cells? Is recovery slower in CHIR99021-induced cells?

Reviewer 2

Advance summary and potential significance to field

The manuscript by Yang et al. describes a study on the dynamic expression pattern of FZD5 in human PSC-derived neural progenitor cells, and the discovery that use of a novel FZD5 agonist can substitute for GSK3 inhibition in hPSC protocols to generate ventral midbrain dopamine neurons for transplantation. The study is very thorough and includes an impressive range of techniques to compare DA cells produced with FZD5 agonist (F5L6.13) to DA cells produced with GSK3i (CHI99021).
The two cultures are compared using ICC, qRT-PCR, transplantation to 6-OHDA animals, electrophysiology, bulk RNAseq and scRNAseq. Overall, the conclusion from the data is that there is

© 2024. Published by The Company of Biologists under the terms of the Creative Commons Attribution License (https://creativecommons.org/licenses/by/4.0/).

not much – if any – biologically significant difference between the GSK3i-patterned and F5L6.13-patterned cells. This means that F5L6.13 can readily substitute for GSK3i in in vitro VM protocols. The study is of interest to the field, and it is well-performed and thorough, for which it deserves publication. A few points however need amendments prior to publication.

Comments for the author

- The link between Figure 1 and Figures 3-6 is unclear and confusing. It is shown in Fig. 1 that FZD5 is not expressed at all in midbrain cells, and that it is only expressed in Forebrain. Yet, in the Results section it is stated that the data from Figure 1 supported the hypothesis that activation of FZD 5 can induce VM patterning. This link does not make much sense and should be revised. Also:
  o Figure 1 appears to be showing data from dorsal midbrain cells, whereas figures 3-6 are dealing with ventral midbrain cells? If so please specify this in Fig. 1. Given the large focus on VM cells later in the protocol, Figure 1 should also include assessment of FZD5 expression in ventral populations (ventral FB ventral MB and ventral HB). It's quite possible you might find here that FZD 5 is expressed in ventral midbrain but not dorsal midbrain cells.

- Please mark EN1 in the volcano plot of Fig. 3H as this is an important marker of caudal VM cells.

- It is unclear how many biological replicate samples were used for the scRNAseq experiment as no n-value is stated in the text or figure. Was only 1 replicate used for each condition? If this is the case, then the authors cannot give any firm conclusions on differences between the conditions based on DEGs.

Although the authors conclude that there is a slightly higher maturation signature in the F5L6.13-treated cells, which could very likely just be a batch-to-batch variation in the data of a single experiment (just as if having n=1 on a qRT-PCR).
In summary, please specify the biological replicate n-value in the scRNAseq exprements, and remove any concluding statements if only one biological replicate was used.

- Please include a volcano plot for the DEGs identified in the scRNAseq experiment.

- Consider if the title of the manuscript should be changed, given that a major focus of the paper is to generate VM cells with the FZD5 agonist.

First revision

Author response to reviewers' comments

Reviewer 1 Advance Summary and Potential Significance to Field:
Yang and colleagues have uncovered a novel mechanism of WNT signalling regulation via differential expression of WNT receptors, Frizzleds (FZDs), during pluripotent stem cell differentiation. They take advantage of this new knowledge to specifically active one (FZD5) of the 10 FZD receptors using a tetravalent antibody they have developed. The results of specifically activating FZD5 instead of generically activating WNT signalling GSK3beta inhibitors were tremendous. Specification of true substantia nigra neurons was significantly increased and were of greater maturity. This is a major leap forward in the understanding of ventral mibrain patterning, and provides a practical tool to improve differentiation of transplantable cells for Parkinson's.

Reviewer 1 Comments for the Author:
Yang and colleagues have uncovered a novel mechanism of WNT signalling regulation via differential expression of WNT receptors, Frizzleds (FZDs), during pluripotent stem cell differentiation. They take advantage of this new knowledge to specifically active one (FZD5) of the 10 FZD receptors using a tetravalent antibody they have developed. The results of specifically activating FZD5 instead of generically activating WNT signalling GSK3beta inhibitors were tremendous. Specification of true substantia nigra neurons was significantly increased and were of

© 2024. Published by The Company of Biologists under the terms of the Creative Commons Attribution License (https://creativecommons.org/licenses/by/4.0/).

greater maturity. This is a major leap forward in the understanding of ventral mibrain patterning, and provides a practical tool to improve differentiation of transplantable cells for Parkinson's.

We are grateful that Reviewer 1 thinks our work constitutes a major leap forward in the field and that it provides new reagent that may have a clinical impact.

Some minor comments to address include:
1. It's not accurate to state “OTX2 and LDB1 are part of a gene regulatory network restricting FZD5 expression within anterior neural progenitors” since the deletion of OTX1 or LDB1 causes a failure of differentiation, and therefore a downstream effect could be loss of FZD5 expression I.e. it may not have anything to do with a ‘gene regulatory network’.

We agree with the reviewer. We recognize that the loss of FZD5 expression may be a downstream effect caused by the loss of OTX2 and LDB1. We have changed the wording in the manuscript to “FZD5 cell surface expression is dynamically regulated across the anterior-posterior axis and FZD5 expression is dependent on anterior forebrain regulators OTX2 and LDB1”.

2. 269 up and 72 down genes when F5L6.13 is compared to CHIR99021 mDA cells for bulk RNAseq. Can KEGG pathway or GO analysis been performed on these gene lists?

We appreciate the reviewer’s comment. We have performed GO and KEGG pathway analyses on the 269 upregulated and 72 downregulated genes when comparing F5L6.13 to CHIR99021 mDA cells for bulk RNAseq. The top 10 enriched GO pathways and KEGG pathways (only present in the upregulated gene set) for each gene list are presented in the table below. We observed overlapping gene ontology enrichment and this may be attributed to the general stage of neuronal differentiation. As such, we focused our analysis on genes explicitly associated with midbrain dopaminergic neuron differentiation.

Upregulated genes

	term_name	term_id	adjusted_p_value	term_size	intersection_size
GO:BP	generation of neurons	GO:0048699	1.07E-27	1485	79
GO:BP	nervous system development	GO:0007399	4.28E-27	2513	101
GO:BP	system development	GO:0048731	6.96E-27	3951	127
GO:BP	multicellular organismal process	GO:0032501	1.18E-25	7648	178
GO:BP	neuron differentiation	GO:0030182	2.55E-25	1404	74
GO:BP	multicellular organism development	GO:0007275	1.57E-24	4610	134
GO:BP	neurogenesis	GO:0022008	2.30E-24	1708	80
GO:BP	neuron development	GO:0048666	1.68E-23	1142	65
GO:BP	developmental process	GO:0032502	3.57E-23	6414	158
GO:BP	anatomical structure development	GO:0048856	5.89E-23	5862	150
KEGG	Insulin secretion	KEGG:04911	0.000313657	86	9
KEGG	Arrhythmogenic right ventricular cardiomyopathy	KEGG:05412	0.009995558	77	7
KEGG	Axon guidance	KEGG:04360	0.023576054	181	10
KEGG	Oxytocin signaling pathway	KEGG:04921	0.032000021	154	9

Downregulated genes:

© 2024. Published by The Company of Biologists under the terms of the Creative Commons Attribution License (https://creativecommons.org/licenses/by/4.0/).

	term_name	term_id	adjusted_p_value	term_size	intersection_size
GO:BP	growth	GO:0040007	0.000918615	930	14
GO:BP	neuron differentiation	GO:0030182	0.004909727	1404	16
GO:BP	developmental process	GO:0032502	0.005984467	6414	37
GO:BP	cell-cell adhesion	GO:0098609	0.006344799	939	13
GO:BP	multicellular organismal process	GO:0032501	0.006410821	7648	41
GO:BP	developmental growth	GO:0048589	0.006492152	655	11
GO:BP	cell morphogenesis	GO:0000902	0.007807014	957	13
GO:BP	cell migration	GO:0016477	0.009271066	1475	16
GO:BP	anatomical structure morphogenesis	GO:0009653	0.010089825	2683	22
GO:BP	generation of neurons	GO:0048699	0.01010776	1485	16

3. What's the evidence that "Cellular heterogeneity inevitably hampers engraftment efficiency or the generation of functional cells"? As long as some correctly patterned cells are in the mixture, the transplants promote recovery.

We agree with the reviewer that while transplants with a mixture of correctly patterned and improperly patterned cells may still promote some degree of recovery. However, the overall engraftment efficiency and functionality of the transplanted cells is likely to be suboptimal due to the presence of non-functional or improperly differentiated cells. We acknowledge the complexity of these processes and the potential for functional recovery, even with a mixed population. However, our statement aims to highlight the importance of minimizing cellular heterogeneity to maximize the therapeutic potential of transplanted cells. We have revised the text in the manuscript to better indicate this.

4. Set-up a ShinyApp of all scRNAseq data for others to easily analyse data.

As suggested by the reviewer we have setup a ShinyApp for readers to access the data in a convenient way. The ShinyApp can be accessed by using the following link: https://andyydh.shinyapps.io/scRNA_VM_progenitor/

© 2024. Published by The Company of Biologists under the terms of the Creative Commons Attribution License (https://creativecommons.org/licenses/by/4.0/).

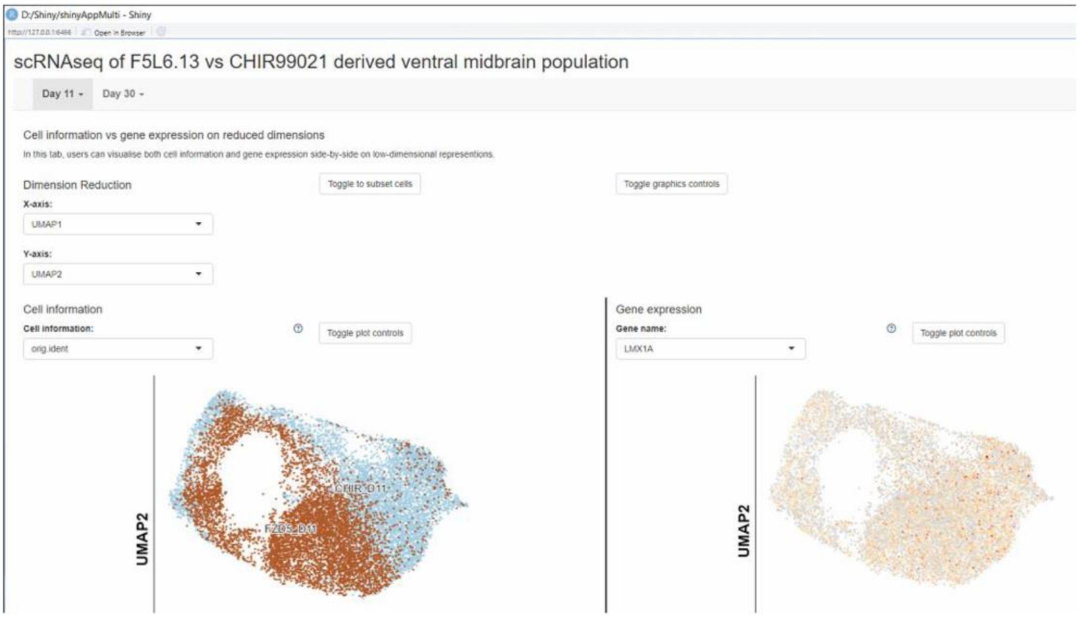

5. Have CHIR99021-induced mDA cells been transplanted in the 6-OHDA model and how does functional recovery compare to F5L6.13-induced mDA cells? Is recovery slower in CHIR99021-induced cells?

We appreciate the reviewer comment. We have not directly compared the transplantation outcomes of CHIR99021-induced mDA cells with F5L6.13-induced cells in the 6-OHDA model. However, the timepoint at which F5L6.13-induced mDA cells exhibit improved motor function in the 6-OHDA animal rat model is consistent with established literatures on CHIR99021-differentiated mDA cells, which typically requires approximately 12 weeks for neuronal maturation to exhibit behavioral benefits in the 6-OHDA model (Kriks et al., 2011; Kirkeby et al., 2012). Whether functional recovery is observed with only a fraction of functional cells is currently unknown. A quantitative comparison of these two cell products is beyond the overall goal of this study.

Reviewer 2:

Advance Summary and Potential Significance to Field: The manuscript by Yang et al. describes a study on the dynamic expression pattern of FZD5 in human PSC-derived neural progenitor cells, and the discovery that use of a novel FZD5 agonist can substitute for GSK3 inhibition in hPSC protocols to generate ventral midbrain dopamine neurons for transplantation. The study is very thorough and includes an impressive range of techniques to compare DA cells produced with FZD5 agonist (F5L6.13) to DA cells produced with GSK3i (CHI99021). The two cultures are compared using ICC, qRT-PCR, transplantation to 6-OHDA animals, electrophysiology, bulk RNAseq and scRNAseq. Overall, the conclusion from the data is that there is not much – if any – biologically significant difference between the GSK3i-patterned and F5L6.13-patterned cells. This means that F5L6.13 can readily substitute for GSK3i in in vitro VM protocols. The study is of interest to the field, and it is well-performed and thorough, for which it deserves publication. A few points however need amendments prior to publication.

We appreciate the comment of the reviewer and the overall positive appreciation of our study. We agree that, at a high level, the two cell products (differentiated using CHIR99021 or F5L6.13) are overall similar. We showed that there are small but may be important differences. Further studies will be needed to evaluate whether it will result in better outcome when transplanted in *in vivo* models.

Reviewer 2 Comments for the Author:
`

© 2024. Published by The Company of Biologists under the terms of the Creative Commons Attribution License (https://creativecommons.org/licenses/by/4.0/).

- The link between Figure 1 and Figures 3-6 is unclear and confusing. It is shown in Fig. 1 that FZD5 is not expressed at all in midbrain cells, and that it is only expressed in Forebrain. Yet, in the Results section it is stated that the data from Figure 1 supported the hypothesis that activation of FZD 5 can induce VM patterning. This link does not make much sense and should be revised. Also: oFigure 1 appears to be showing data from dorsal midbrain cells, whereas figures 3-6 are dealing with ventral midbrain cells? If so please specify this in Fig. 1. Given the large focus on VM cells later in the protocol, Figure 1 should also include assessment of FZD5 expression in ventral populations (ventral FB, ventral MB and ventral HB). It's quite possible you might find here that FZD 5 is expressed in ventral midbrain but not dorsal midbrain cells.

We appreciate the reviewer's comments, which prompted us to revisit and modify the manuscript. In the revised text, we have clarified the role of FZD5 protein cell surface expression and activation during the neural induction phase of hPSC, particularly emphasizing its temporal control during Wnt/ß-catenin pathway activation.

FZD5 expression at the cell surface starts to elevate at day 2 of neural differentiation (Fig 1C). This expression provides a unique spatiotemporal control of Wnt activation with the FZD5 selective F5L6.13 agonist during posterior patterning (as opposed to activating FZD2 or FZD7 that are constitutively expressed at all time points on the cell surface). In current ventral midbrain patterning protocols, CHIR99021 is added from D0-9 of differentiation to activate Wnt/ß-catenin signaling for posteriorization (Kirkbey et al., 2012). In our protocol, FZD5 stimulation starts at day 2, as soon as FZD5 expression is induced and thus become responsive to posteriorization signals, thereby promoting midbrain patterning. As soon as cells adopt a midbrain fate, FZD5 cell surface expression is downregulated and likely prevents further posteriorization.

Following the reviewer's suggestion, we performed additional experiments and the results confirm that FZD5 is uniquely expressed in the anterior-patterned NPC in both dorsal and ventral differentiation conditions. We have incorporated this data in Figure 1E and added this phrase in the results section "Importantly, FZD5 cell surface expression remained upregulated in anterior neural progenitors in ventral patterning conditions (Fig. 1E), indicating its potential as a target for ventral midbrain patterning using a selective FZD5 FLAg antibody agonist, given its early upregulation during the neural induction phase."

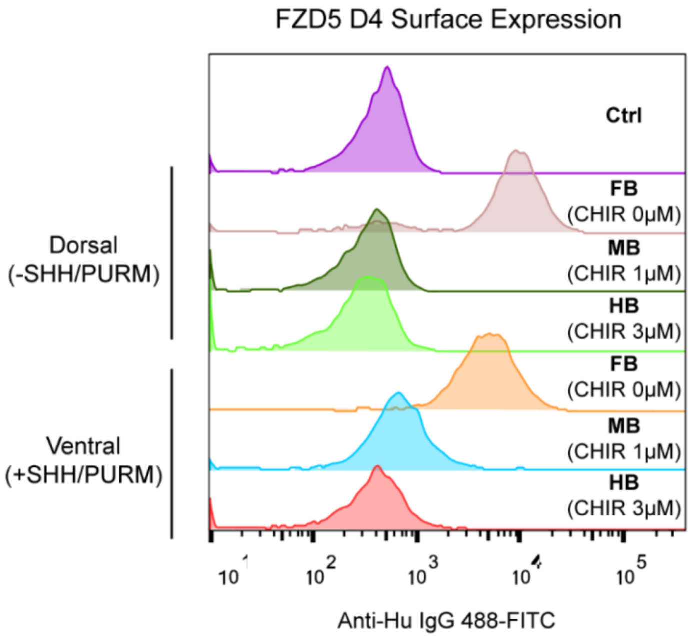

-Please mark EN1 in the volcano plot of Fig. 3H as this is an important marker of caudal VM cells.

We have now plotted EN1 in the volcano plot.

-It is unclear how many biological replicate samples were used for the scRNAseq experiment as

© 2024. Published by The Company of Biologists under the terms of the Creative Commons Attribution License (https://creativecommons.org/licenses/by/4.0/).

no n-value is stated in the text or figure. Was only 1 replicate used for each condition? If this is the case, then the authors cannot give any firm conclusions on differences between the conditions based on DEGs. Although the authors conclude that there is a slightly higher maturation signature in the F5L6.13-treated cells, which could very likely just be a batch-to-batch variation in the data of a single experiment (just as if having n=1 on a qRT-PCR). In summary, please specify the biological replicate n-value in the scRNAseq exprements, and remove any concluding statements if only one biological replicate was used.

We used a single biological replicate for each condition in our scRNAseq study. In single- cell sequencing experiments it is not uncommon to use one biological replicate due to unique challenges with this technique (cost and technical complexities). We recognize the limitations of relying on a single replicate, but it is important to keep in mind that the comparison was performed in parallel with the same cells during the differentiation and were processed and analyzed simultaneously in a multiplex sequencing format using 10x CellPlex. We have revised the manuscript to indicate that this was performed on one biological sample.

-Please include a volcano plot for the DEGs identified in the scRNAseq experiment.

Thank you to the reviewer for this suggestion. We have incorporated a volcano plot (Figure 5F) illustrating the DEGs identified from comparing cluster 0 and cluster 1,2,5 in the scRNAseq experiment between day 11 CHIR99021-induced cells and F5L6.13- differentiated cells. Regarding the DEGs identified in the HiDDEN analysis comparing DA neurons, we have emphasized the subset of genes associated with neurodevelopment in dot plots, specifically in Figure S6J, showcasing the differences between CHIR_L0, F5L6_L0 (CHIR-like cells), and F5L6_L1. This approach allows us to highlight the uniqueness of F5L6_L1 cells compared to both CHIR_L0 and F5L6_L0 and offers a more informative representation of the transcriptional differences in the cell population. We will be including the list of DEGs identified comparing L0 to L1 as a supplemental table (Table S3) in the manuscript.

-Consider if the title of the manuscript should be changed, given that a major focus of the paper is to generate VM cells with the FZD5 agonist.

We appreciate this suggestion by the reviewer. Here is our suggestion for a new title: Exploiting Spatiotemporal Regulation of FZD5 during Neural Patterning for Efficient Ventral Midbrain Specification.

Second decision letter

MS ID#: DEVELOP/2023/202545

MS TITLE: Exploiting Spatiotemporal Regulation of FZD5 during Neural Patterning for Efficient Ventral Midbrain Specification.

AUTHORS: Andy Yang, Rony Chidiac, Emma Russo, Hendrik Steenland, Quinn Pauli, Robert Bonin, Levi L Blazer, Jarrett J Adams, Sachdev S Sidhu, Alexandrina Goeva, Ali Salahpour, and Stephane Angers

I have now received all the referees reports on the above manuscript, and have reached a decision. The referees' comments are appended below, or you can access them online: please go to BenchPress and click on the 'Manuscripts with Decisions' queue in the Author Area.

The overall evaluation is positive and we would like to publish a revised manuscript in Development, provided that the referees' comments can be satisfactorily addressed. Please attend to Reviewer 2's concern about the conclusions drawn from scRNAseq experiment, I agree that moderating these conclusions would be appropriate. If you do not agree with these criticisms and suggestions please explain clearly why this is so.

© 2024. Published by The Company of Biologists under the terms of the Creative Commons Attribution License (https://creativecommons.org/licenses/by/4.0/).

Reviewer 1

Advance summary and potential significance to field

All comments addressed in full.

Comments for the author

Revised manuscript is addresses all comments in full.

Reviewer 2

Advance summary and potential significance to field

This is a revised version of the manuscript DEVELOP/2023/202545.

Comments for the author

The authors have addressed most of my concerns, although I remain concerned about the extensive amounts of conclusions which are derived from scRNAseq experiment in Fig 5 and 6 with n=1. While it is generally acceptable to draw conclusions relating to cell type heterogeneity, cell type-specific markers and cellular trajectories in a representative culture from an n=1 scRNAseq experiments, it is in contrast not acceptable to draw conclusions on treatment effects (in this case CHIR versus F5L6.13) based on a single replicate – no matter if this is done by ICC, qRT-PCR or scRNAseq. There are good methods available to include several biological replicates in a single scRNAseq experiment
– i.e. through cell hashing
– without increasing the cost of the experiment. However, as this has not been done here, it must be very clearly apparent both in the text and in the legend that these data are not statistically relevant:

- It must be stated in the figure legends to Fig 5 and 6 that these data are based on an n=1 experiment.

- It must be clearly noted in the schematic in Fig 5A that the experiment was done with n=1

- Remove the word "significantly" from this sentence. "Specifically, CHIR99021 patterned cells were overrepresented in clusters 1, 2, and 5 but significantly underrepresented in cluster 0 when compared to F5L6.13 patterned cells."

- The conclusion statement for Fig 5 should be moderated to include an additional sentence stating the limitations of the data – a suggestion: "… with individual cells showing differential regulation of several signaling pathways known to involve GSK3 activity. However, it should be noted that this analysis is based on a sample size of n=1, and analysis of further replicates is needed for confirmation."

- Also the conclusion statement for figure 6 should be moderated – a suggestion: "Overall, the data indicates that F5L6.13 is equally efficient as CHIR99021 in directing neural progenitors toward the DA neuron fate and the HiDDEN analysis suggest that a subpopulation of DA neurons generated by F5L6.13 aligns more closely with the anatomical origin of DA neurons, however, analysis of further replicates is needed for confirmation."

Second revision

Author response to reviewers' comments

© 2024. Published by The Company of Biologists under the terms of the Creative Commons Attribution License (https://creativecommons.org/licenses/by/4.0/).

Reviewer 1 Advance Summary and Potential Significance to Field:
All comments addressed in full.

Reviewer 1 Comments for the Author:
Revised manuscript is addresses all comments in full.

Reviewer 2 Comments for the Author:
The authors have addressed most of my concerns, although I remain concerned about the extensive amounts of conclusions which are derived from scRNAseq experiment in Fig 5 and 6 with n=1. While it is generally acceptable to draw conclusions relating to cell type heterogeneity, cell type-specific markers and cellular trajectories in a representative culture from an n=1 scRNAseq experiments, it is in contrast not acceptable to draw conclusions on treatment effects (in this case CHIR versus F5L6.13) based on a single replicate - no matter if this is done by ICC, qRT-PCR or scRNAseq. There are good methods available to include several replicates in a single scRNAseq experiment - i.e .through cell hashing - without increasing the cost of the experiment. However, as this has not been done here, it must be very clearly apparent both in the text and in the legend that these data are not statistically relevant:

Thank you for the reviewer's comment. We have revised the manuscript to clarify that the scRNA experiment was performed using one biological replicate and have modified the concluding statement in line with the reviewer's suggestions.

-It must be stated in the figure legends to Fig 5 and 6 that these data are based on an n=1 experiment.

The figure legend in Fig 5 now reads "5A. Schematic of the workflow in 10x Cellplex pipeline labelling the differentiated cells with CMOS for barcoding prior to sample processing and sequencing. This experiment was performed using one biological replicate in parallel." and Fig 6. reads "A. UMAP plot showing clustering of the day 30 single cells from one biological replicate".

-It must be clearly noted in the schematic in Fig 5A that the experiment was done with n=1

The schematic in Fig 5A is modified to show N=1.

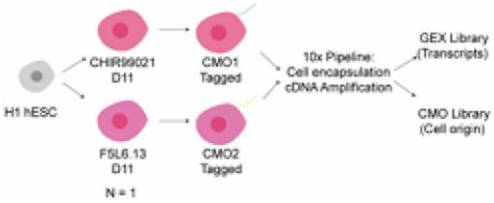

- Remove the word "significantly" from this sentence. "Specifically, CHIR99021 patterned cells were overrepresented in clusters 1, 2, and 5 but significantly underrepresented in cluster 0 when compared to F5L6.13 patterned cells."

The sentence is revised accordingly.

-The conclusion statement for Fig 5 should be moderated to include an additional sentence stating the limitations of the data - a suggestion: "... with individual cells showing differential regulation of several signaling pathways known to involve GSK3 activity.
However, it should be noted that this analysis is based on a sample size of n=1, and analysis of further replicates is needed for confirmation."

Thank you for the reviewer's suggestion. We have now revised the manuscript accordingly: "Consistent with the results above, F5L6.13-patterned progenitors exhibited an increase in the proportion of cell clusters exhibiting modulation of Notch signaling while CHIR99021- patterned progenitors gave rise to increased proportion of clusters with modulation of MAPK and mTOR pathways (Fig. 5H). This suggests that individual cells derived from the two treatments show differential regulation of several signaling pathways known to involve GSK3. Overall, our single-cell

© 2024. Published by The Company of Biologists under the terms of the Creative Commons Attribution License (https://creativecommons.org/licenses/by/4.0/).

transcriptome profiling of day 11 cell populations shows that using CHIR99021- and F5L6.13-patterned NPC gives rise to VM fate with high purity." In addition, we have indicated in the materials and methods, in the text and the figure legend that the experiment is performed using one biological replicate and we highlighted the limitation of this approach and note that further replicates are needed for confirmation in the discussion.

-Also the conclusion statement for figure 6 should be moderated – a suggestion: "Overall, the data indicates that F5L6.13 is equally efficient as CHIR99021 in directing neural progenitors toward the DA neuron fate and the HiDDEN analysis suggest that a subpopulation of DA neurons generated by F5L6.13 aligns more closely with the anatomical origin of DA neurons, however, analysis of further replicates is needed for confirmation."

Thank you for the reviewer's suggestion. We have now revised the manuscript accordingly: "Gene ontology (GO) enrichment analysis of the 394 DE genes enriched in F5L6_L1 DA neurons revealed that this subpopulation is enriched in GO terms of substantia nigra development and neuronal differentiation processes (Fig. 5K, S6J). This analysis suggests that a subpopulation of DA neurons generated by F5L6.13 aligns more closely with the anatomical origin of DA neurons. Overall, F5L6.13 is equally efficient as CHIR99021 in directing neural progenitors toward the DA neuron fate." In addition, we have indicated in the materials and methods and the figure legend that the experiment is performed using one biological replicate and we highlighted the limitation of this approach and note that further replicates are needed for confirmation in the discussion.

Third decision letter

MS ID#: DEVELOP/2023/202545

MS TITLE: Exploiting Spatiotemporal Regulation of FZD5 during Neural Patterning for Efficient Ventral Midbrain Specification.

AUTHORS: Andy Yang, Rony Chidiac, Emma Russo, Hendrik Steenland, Quinn Pauli, Robert Bonin, Levi L Blazer, Jarrett J Adams, Sachdev S Sidhu, Aleksandrina Goeva, Ali Salahpour, and Stephane Angers
ARTICLE TYPE: Research Article

I am happy to tell you that your manuscript has been accepted for publication in Development, pending our standard ethics checks.

© 2024. Published by The Company of Biologists under the terms of the Creative Commons Attribution License (https://creativecommons.org/licenses/by/4.0/).
